# Primary healthcare, climate-related health impacts and intercultural dialogue: bibliometric evidence of a fragmented research field

**DOI:** 10.3389/frhs.2026.1909093

**Published:** 2026-09-30

**Authors:** Nidia Ponte, Fátima Alves, Diogo Guedes Vidal

**Affiliations:** 1 Department of Social Sciences and Management, Universidade Aberta, Lisbon, Portugal; 2 Centre for Functional Ecology - Science for People & the Planet (CFE), TERRA Associate Laboratory, Department of Life Sciences (DCV), University of Coimbra (UC), Coimbra, Portugal

**Keywords:** bibliometric analysis, climate change and health, climate-related health impacts, cultural competence, culturally diverse populations, health equity, intercultural communication, primary healthcare

## Abstract

Primary healthcare, climate-related health impacts, and intercultural dialogue are increasingly relevant to health equity and health service adaptation, yet it remains unclear whether these domains form an integrated research field or remain partially connected literatures. This systematic bibliometric analysis addresses this question by mapping how primary healthcare, climate-related health impacts, and intercultural dialogue appear in the literature. For this purpose, records were retrieved from Scopus, Web of Science Core Collection, PubMed, SciELO, RCAAP and Google Scholar, with additional records identified through exploratory searching and the lead author's library. Records published between 1 January 2007 and 31 March 2026 in English, Portuguese or Spanish were included if they addressed one or more domains of the pre-specified Population–Exposure–Outcome framework: primary healthcare physicians and culturally diverse adult patients (Population); direct or indirect health impacts of climate change (Exposure); and intercultural dialogue strategies relevant to the primary-care doctor–patient relationship (Outcome). Records were managed in Rayyan and Microsoft Excel. Descriptive analyses characterised search yield, source contribution, screening attrition, document profile, language, geographical context, publication venues and citation visibility. VOSviewer supported keyword co-occurrence, temporal overlay and co-authorship mapping. These analyses were conducted on the full-text assessed corpus, distinct from the smaller subset retained for qualitative synthesis. Searches identified 19,211 records: 13,816 after deduplication; 801 reports sought for retrieval, of which 56 could not be retrieved. This left 745 reports for full-text assessment, none of which met the complete Population + Exposure + Outcome configuration; the strongest partial intersection comprised 64 reports addressing only Population and Outcome. Keyword co-occurrence analysis reinforced this pattern: a consolidated thematic core centred on access to healthcare, cultural competence, primary healthcare, communication, migrant populations and sociocultural diversity, whereas climate-related terms remained more peripheral, more recent and less structurally embedded. Taken together, these findings indicate that primary healthcare, climate-related health impacts and intercultural dialogue remain adjacent rather than integrated bodies of literature, with cultural competence and migrant-health research far more consolidated than the climate-health dimension. Closing this gap requires research that directly examines how climate-sensitive health risks are recognised, communicated and negotiated in intercultural primary-care encounters, to inform both clinical training and climate-adaptation policy.**Systematic Review Registration**: https://www.crd.york.ac.uk/PROSPERO/view/CRD420251038912, PROSPERO CRD420251038912.

## 1 Introduction

Climate change is widely recognised as a major threat to public health. Its effects are evident in heat-related illnesses, respiratory and cardiovascular diseases, vector-borne infections, and the health impacts of extreme weather events (1, 2). Beyond direct physiological harm, climate change also contributes to food insecurity, displacement, environmental degradation, mental health challenges, and disruption of health and social systems (3–6). These impacts are unevenly distributed, with individuals and communities already facing social, cultural, linguistic, economic, or territorial vulnerability experiencing greater barriers to protection, timely care, and adaptation (7, 8).

This vulnerability is compounded where health systems serve increasingly diverse sociocultural populations. In clinical encounters, sociocultural background shapes symptom description, risk perception, trust-building, and how professionals and patients negotiate recommendations. Intercultural communication, cultural competence, cultural safety, and equity-oriented care are central to healthcare quality. These factors are especially important where language barriers, migration histories, minority status, or unequal access to services affect patient-provider relationships (9–11).

Primary care is particularly important in this context. As the first point of contact and a setting for ongoing relationships with individuals, families, and communities, it plays a key role in recognising climate-sensitive health risks, supporting prevention and adaptation, and communicating risk in ways that are both clinically meaningful and socially understandable (12–14). This review focuses specifically on general and family medicine physicians, given their well-documented role in communicating climate-related risks to patients and in leading practice- and organisational-level responses to climate change (12–14), including recent evidence that general practitioners already engage directly with patients on this topic in primary care (15). Effective responses to climate-related health impacts require not only technical preparedness but also communication practices that consider culturally situated experiences of vulnerability, resilience, illness, and care.

Despite the clear relevance of such communication practices, it remains unclear how far the existing literature reflects them. Although climate change and health, intercultural communication, and primary care have each generated substantial bodies of literature, the extent to which these domains connect remains difficult to characterise. Relevant studies may be dispersed across disciplines, databases, vocabularies and publication venues, making it challenging to identify how evidence on culturally responsive climate-health adaptation in primary care is organised. Most studies focus on access to primary healthcare, cultural competence, health communication, and migrant/diverse populations (16–21). Intercultural dialogue is primarily addressed in relation to cultural competence in care provision for migrant and refugee populations (18, 21, 22). Recent reviews highlight the literature's fragmented nature: while numerous studies examine pairwise relationships (e.g., climate–health and migration–health), few, if any, address the intersection of all three dimensions simultaneously (23, 24).

This study aimed to map the literature at the intersection of primary care, intercultural communication and climate-related health impacts, and to characterise how this research landscape is organised across publication patterns, citation visibility, collaboration structures and thematic co-occurrence. Bibliometric mapping is well suited to this purpose because it can clarify the distribution, structure, and thematic relationships of a research field.

## 2 Materials and methods

### 2.1 Study design and registration

This study is a systematic bibliometric review combining descriptive performance analysis and science mapping. The aim was to examine how the retrieved literature is distributed, structured and fragmented across three conceptual domains: primary healthcare, climate-related health impacts and intercultural dialogue. We used bibliometric methods because the topic is broad, interdisciplinary, and dispersed across several databases, vocabularies, and publication venues (25, 26).

We first submitted the broader review protocol to PROSPERO (CRD420251038912) and later expanded and registered it on the Open Science Framework (OSF: 10.17605/OSF.IO/AXYDW) (27, 28). The protocol followed PRISMA-P and PRISMA-S principles, and we reported this bibliometric component in line with BIBLIO guidance for bibliometric reviews of biomedical literature (26, 29–31). The OSF registration was updated to align the Population, Exposure, and Outcome definitions with the PROSPERO record and to clarify the context criterion; these amendments were editorial and did not change the research questions or analytical approach. Because screening and curation extended beyond the initial timetable, the search was updated on 1 April 2026.

### 2.2 Eligibility framework and corpus definition

Box 1 shows the pre-specified Population–Exposure–Outcome framework. The Population domain comprised primary healthcare physicians, particularly general and family medicine physicians, as well as adult patients from diverse sociocultural backgrounds. The Exposure domain encompassed direct and indirect health impacts of climate change, including heat stress, extreme weather events, food insecurity, forced displacement and other forms of ecological vulnerability. The Outcome domain comprised intercultural dialogue strategies, including cultural competence education and equity-oriented approaches relevant to climate adaptation, community resilience and the primary-care doctor–patient relationship.

BOX 1 **Population–exposure–outcome framework used to guide eligibility assessment**

**Table:** 

Domain	Operational definition
Population (P)	Primary healthcare physicians, particularly general and family medicine physicians (general practitioners), and adult patients (aged 18 and over) from diverse sociocultural backgrounds.
Exposure (E)	Impacts of climate change on health, whether direct or indirect, include heat stress, extreme weather events, food insecurity, forced displacement, and other forms of ecological vulnerability. This includes consideration of how these exposures are recognised and responded to through face-to-face communication between healthcare professionals and patients.
Outcome (O)	Intercultural dialogue strategies developed, and/or implemented—including cultural competence education and equity-oriented approaches—that contribute to climate adaptation and community resilience and are relevant to the doctor–patient relationship in primary healthcare or demonstrably transferable to it.
This box reproduces the PEO eligibility framework pre-specified in the registered PROSPERO and OSF protocols (Ponte et al., 2025a, 2025b), with minor editorial formatting changes.	

Eligible records included peer-reviewed journal articles, empirical studies, reviews, conceptual or theoretical papers, books and book chapters, conference papers, doctoral theses, master's dissertations, research reports and technical documents, if they contained substantive material relevant to the review scope. Editorials, letters, errata, short comments, press pieces and documents without sufficient analytical content were excluded. Records were limited to English, Portuguese and Spanish publications from 1 January 2007 to 31 March 2026.

The same pre-specified Population–Exposure–Outcome framework guided screening, full-text assessment, and conceptual classification throughout the review. Separate conceptual eligibility criteria were not applied to the bibliometric corpus and the qualitative subset because they were not independent analytical sets: the 64-report qualitative subset was nested within the 745-report full-text-assessed corpus used for bibliometric analysis, as shown in the PRISMA flow diagram (Figure 1). The bibliometric corpus included all reports successfully retrieved and advanced to full-text assessment (*n* = 745). Full-text assessment enabled consistent classification of each report in relation to the P, E, and O domains, including single-domain records, the P + O partial intersection, and broader PEO mismatch. Retaining these records for bibliometric mapping was intentional, as this component examined how the three predefined domains were distributed, connected, or separated across the retrieved literature. Within this 745-report corpus, 64 reports were classified as P + O and retained for subsequent qualitative synthesis. No report met the complete P + E + O configuration, and we identified no genuine P + E or E + O intersections under the predefined domain definitions. Reports not retained for qualitative synthesis remained part of the bibliometric corpus; their PEO classification contributed directly to mapping the conceptual structure surrounding the review question. This broader analytical corpus is necessarily more heterogeneous than the P + O subset and may therefore increase thematic dispersion and reduce apparent connectivity within the co-authorship network; this was considered when interpreting the observed fragmentation.

**Figure 1:**
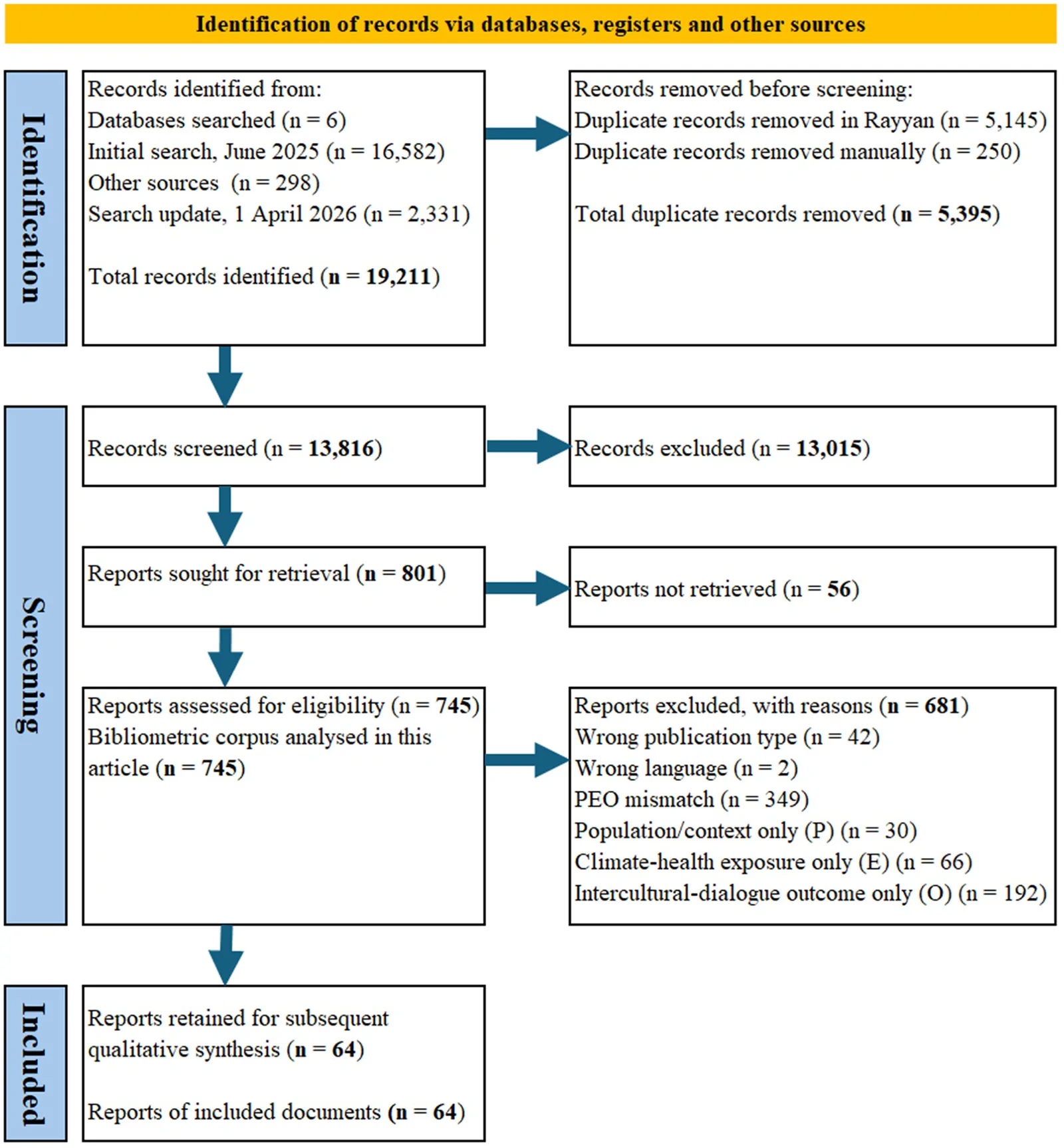
PRISMA 2020 flow diagram adapted for the construction of the bibliometric corpus. For the purposes of this bibliometric article, the analytical corpus comprises the 745 reports assessed for eligibility. The 64 studies reported in the final PRISMA box represent the subset retained after full-text eligibility assessment for the subsequent qualitative review. Adapted from the PRISMA 2020 flow diagram template by Page et al. (31), licensed under CC BY 4.0.

### 2.3 Information sources and search strategy

Searches were conducted in Scopus, Web of Science Core Collection, PubMed, SciELO, RCAAP and Google Scholar. These sources were selected to balance biomedical coverage, social-science breadth, interdisciplinary retrieval, linguistic diversity and access to grey literature. Additional records were included under “Other sources” when they had been identified during exploratory searching or were already present in the author's bibliographic library and appeared potentially relevant to the review scope.

The search strategy focused on the three PEO domains. Synonyms within each domain were combined using OR, and conceptual blocks were combined using AND. Because preliminary scoping suggested that the literature was distributed across partial intersections, the strategy did not rely only on the full P + E + O combination. Where database functionality allowed, we used four combinations: P + E + O, P + E, P + O, and E + O. As detailed in the Results, full-text assessment did not identify records meeting a genuine P + E or E + O intersection under these domain definitions. The initial searches were conducted in June 2025, and the update was conducted on 1 April 2026, capturing records published between 1 June 2025 and 31 March 2026. We adapted search fields and syntax to each source. The full database-specific search strategies, field restrictions, filters, and platform-specific adaptations are available in the registered OSF protocol; Table 1 summarises search yields and source contribution. For Google Scholar, given the platform's limited advanced-search functionality and lack of a stable, exportable retrieval order, results for each language query (English, Portuguese and Spanish) were screened in descending order of relevance up to the first 100 records per language, consistent with established practice for this database.

**Table 1:** Source contribution, duplicate removal and full-text retrieval yield.

Source	Records identified before deduplication, *n* (%)	Duplicates removed, *n* (% of source records)	Records screened, *n* (%)	Reports sought for retrieval, *n* (% of screened source)	Reports assessed for eligibility, *n* (% of assessed corpus)
RCAAP	130 (0.68%)	6 (4.62%)	124 (0.90%)	7 (5.65%)	7 (0.94%)
Google Scholar	356 (1.85%)	17 (4.78%)	339 (2.45%)	17 (5.01%)	15 (2.01%)
PubMed	303 (1.58%)	63 (20.79%)	240 (1.74%)	33 (13.75%)	32 (4.30%)
Other sources	298 (1.55%)	19 (6.38%)	279 (2.02%)	68 (24.37%)	67 (8.99%)
SciELO	2,292 (11.93%)	450 (19.63%)	1,842 (13.33%)	176 (9.55%)	176 (23.62%)
Web of Science	7,400 (38.52%)	3,965 (53.58%)	3,435 (24.86%)	250 (7.28%)	230 (30.87%)
Scopus	8,432 (43.89%)	875 (10.38%)	7,557 (54.70%)	250 (3.31%)	218 (29.26%)
Total	19,211	5,395 (28.08%)	13,816 (100.00%)	801 (5.80%)	745 (100.00%)

Percentages for records identified and screened are calculated relative to the column total. Percentages for duplicates removed and reports sought for retrieval are calculated within each source. Percentages for reports assessed for eligibility are calculated relative to the full-text corpus assessed for eligibility (*n* = 745). The difference between reports sought for retrieval and reports assessed for eligibility corresponds to reports that could not be retrieved. Duplicate counts by source reflect source redundancy and the operational decision to retain the record with the most complete metadata. Pairwise database overlap was not calculated.

### 2.4 Record management, screening and metadata curation

Records retrieved from formal searches were imported into Rayyan for record management, de-duplication and title-and-abstract screening. Records from sources with limited export functionality were captured manually or through Zotero before being incorporated into the screening workflow. Microsoft Excel was used to maintain screening logs, document decisions, record exclusion reasons and support metadata curation. A structured set of screening labels operationalised the PEO framework, standardised exclusion reasons, and distinguished between single-domain records, partial intersections, and the complete P + E + O configuration.

Duplicate removal combined Rayyan-based detection with manual verification during bibliographic curation. Matching was based primarily on DOI and, where DOI was unavailable, on normalised title, first author and publication year.

The lead author conducted screening at both stages. At title-and-abstract screening, an approximately 10% sample of exclusions was independently reassessed, confirming 1,347 of 1,357 reassessed decisions (99.3%) and reclassifying ten records (0.7%) as potentially eligible. At full-text screening, the lead author reviewed all excluded records a second time before final exclusion; the supervisory team cross-checked a further 10% of exclusion decisions, and the team discussed uncertain or borderline cases before reaching a final decision.

We extracted bibliographic and analytical metadata into a structured Microsoft Excel matrix and manually checked it where necessary. Variables included bibliographic information, source of origin, document type, language, keyword availability, geographical context, thematic domain, PEO coding and screening decision. Missing data were not imputed and were coded as “not specified” or “not available” when they could not be reliably identified.

### 2.5 Bibliometric analysis

Descriptive analyses characterised the corpus by year of publication, source of origin, document type, language, publication venue, geographical context, and thematic domain. Publication trends were examined over time, the most frequent publication venues were assessed for source dispersion, and citation visibility was explored using global citation counts retrieved from Scopus on 9 May 2026. Citation counts were interpreted as indicators of relative visibility, not as measures of methodological quality or definitive intellectual influence. Citation patterns also reveal the structure of scholarly recognition: which authors, works, and perspectives are most frequently acknowledged, how knowledge circulates within the field, and which contributions may remain marginalised or underrepresented.

Science mapping focused on keyword co-occurrence and co-authorship patterns. Keyword co-occurrence analysis identified thematic clusters, central terms, bridging terms, and areas of partial overlap across primary healthcare, intercultural dialogue, and climate-related health. Co-authorship analysis examined collaboration patterns where author metadata were sufficiently complete. Temporal overlay visualisations assessed whether climate-related terms appeared more recently than established terms associated with cultural competence, access to care, communication, and health equity.

We produced network visualisations using VOSviewer, version 1.6.20 (32). Keyword co-occurrence analysis used database keywords as the unit of analysis. Before co-word analysis, a VOSviewer thesaurus was applied to remove administrative tags and source-related labels and to merge minor lexical, spelling and formatting variants. Full counting was used for the main keyword map, with a minimum occurrence threshold of 3, to retain less frequent but conceptually relevant terms in this heterogeneous interdisciplinary corpus. A fractional-counting map was inspected as a sensitivity check. No inferential statistical testing was conducted. Findings are presented as counts, percentages, descriptive trends and bibliometric network outputs. Descriptive tabulations and data checks were conducted in Microsoft Excel, version 365. Screening and de-duplication were managed in Rayyan, version Premium (33), and reference management was supported by Zotero, version 9.0.3 (64-bit) (34).

No formal risk-of-bias or methodological quality assessment was conducted for the bibliometric corpus. The unit of analysis was the bibliographic record and its contribution to mapping the field, not the evidential strength of individual empirical findings. The MMAT-based and reflexive quality-appraisal procedures described in the broader protocol apply to the subsequent qualitative synthesis and will be reported separately.

## 3 Results

The results are organised in five sections. First, we report the search yield and source contribution. Second, we examine the conceptual fragmentation of the full-text assessed corpus using the PEO framework. Third, we characterise the curated bibliometric corpus and its analytical usability. Fourth, we describe temporal, geographical, publication-source, citation and collaboration patterns. Finally, we present the thematic structure and temporal evolution of the study axes through keyword co-occurrence analysis.

### 3.1 Search yield and corpus composition

Figure 1 summarises the search yield across the successive stages of identification, de-duplication, title-and-abstract screening, full-text retrieval and full-text assessment. The initial search, conducted in June 2025, identified 16,880 records across Scopus, Web of Science, PubMed, Google Scholar, RCAAP, SciELO and other sources. The search update conducted on 1 April 2026 identified a further 2,331 records. Overall, we identified 19,211 records before de-duplication.

After removing duplicates, we retained 13,816 unique records for title-and-abstract screening. Of these, we excluded 13,015 records and selected 801 reports for full-text retrieval, representing 5.8% of screened records that progressed to retrieval. Quality-control reassessment of records initially excluded at title-and-abstract screening confirmed most exclusion decisions: 1,357 records were reassessed, 1,347 remained excluded, and 10 were reclassified as potentially eligible. This corresponded to an exclusion confirmation rate of 99.3% and a reclassification rate of 0.7% in the reassessed sample.

Of the 801 reports sought for retrieval, 56 could not be retrieved despite reasonable efforts. The remaining 745 reports were assessed for full-text eligibility and constituted the curated bibliometric corpus used in the main analyses. After full-text assessment, 64 reports were retained for qualitative follow-up analysis, and 681 were excluded. The most frequent reason for full-text exclusion was insufficient alignment with the review's PEO framework.

Source contribution varied substantially across the search process (Table 1). Before deduplication, Scopus was the largest source of records (*n* = 8,432; 43.9%), followed by Web of Science (*n* = 7,400; 38.5%) and SciELO (*n* = 2,292; 11.9%). PubMed, Google Scholar, RCAAP and other sources contributed smaller absolute numbers. Overall, we removed 5,395 duplicate records, corresponding to 28.1% of the records initially identified. Because duplicate records were assigned to the source from which we removed them, these counts reflect both database overlap and the operational rule that retains the record with the most complete metadata, rather than direct pairwise overlap between sources.

After de-duplication, Scopus remained the dominant contributor to the screened dataset (*n* = 7,557; 54.7%), followed by Web of Science (*n* = 3,435; 24.9%) and SciELO (*n* = 1,842; 13.3%). Together, these three sources accounted for 92.9% of screened records. However, source contribution changed when considering progression to full-text retrieval and assessment. Other sources had the highest forwarding rate to full-text retrieval (24.4%), followed by PubMed (13.8%) and SciELO (9.6%). Scopus, despite contributing the largest number of screened records, had the lowest forwarding rate (3.3%), indicating a high volume of records retrieved by the search but excluded after title-and-abstract screening.

The composition of the full-text assessed corpus differed from the screened dataset. Of the 745 reports assessed for eligibility, Web of Science contributed 230 records (30.9%), Scopus 218 (29.3%) and SciELO 176 (23.6%). Other sources contributed 67 records (9.0%), PubMed 32 (4.3%), Google Scholar 15 (2.0%) and RCAAP 7 (0.9%). The difference between the 801 reports sought for retrieval and the 745 reports assessed for eligibility corresponds to reports that could not be retrieved.

Most title-and-abstract exclusions were conceptually driven. The largest exclusion category comprised records addressing only the Exposure dimension (E only; *n* = 4,930; 37.9%), followed by broad PEO mismatch (*n* = 4,482; 34.4%), Outcome only (O only; *n* = 1,651; 12.7%) and Population only (P only; *n* = 1,541; 11.8%). Together, these PEO-related conceptual categories accounted for 12,604 exclusions, corresponding to 96.8% of all title-and-abstract exclusions. Formal, technical or bibliographic reasons, including publication type, language, date limits, missing abstracts or unavailable full text, accounted for a much smaller proportion of exclusions (*n* = 411; 3.2%). This pattern indicates that the search captured substantial literature on individual components of the review question, but comparatively few records articulated the minimum thematic intersection required for eligibility.

### 3.2 Conceptual fragmentation of the full-text assessed corpus

Conceptual fragmentation was examined in the full-text-assessed corpus because these records had available full texts and could therefore be classified more reliably according to PEO alignment. The full-text assessment confirmed that attrition was primarily conceptual rather than formal (Table 2). Of the 745 reports assessed, we retained 64 for qualitative follow-up analysis and excluded 681. Among excluded reports, 637 were excluded for conceptual reasons, representing 93.5% of all full-text exclusions. The largest exclusion category was broad PEO mismatch (*n* = 349; 46.8% of the full-text-assessed corpus), followed by O only (*n* = 192; 25.8%), E only (*n* = 66; 8.9%), and P only (*n* = 30; 4.0%). Formal exclusions were much less frequent, with 42 reports excluded for the wrong publication type and two for the wrong language.

**Table 2:** Conceptual fragmentation of the full-text assessed corpus, *n* = 745.

Full-text assessment category	*n*	%
Included in qualitative synthesis: P + O	64	8.6%
Excluded: PEO mismatch	349	46.8%
Excluded: P only	30	4.0%
Excluded: E only	66	8.9%
Excluded: O only	192	25.8%
Excluded: wrong publication type	42	5.6%
Excluded: wrong language	2	0.3%
Total	745	100.0%

P = primary healthcare physicians and adult patients from diverse sociocultural backgrounds.

E = direct or indirect health impacts of climate change.

O = intercultural dialogue strategies relevant to the primary-care doctor–patient relationship.

Percentages were calculated using the full-text assessed corpus as the denominator (*n* = 745).

Although the search strategy used P + E, P + O and E + O combinations to ensure broad retrieval, full-text assessment did not identify records meeting a genuine P + E or E + O intersection under the domain definitions specified in Box 1; these combinations are therefore not reported as separate categories.

The only configuration retained for qualitative follow-up analysis was P + O. These 64 reports addressed an eligible or transferable primary healthcare context involving health professionals and culturally diverse adult patients, as well as intercultural dialogue, cultural competence education, cultural safety, equity-oriented practice, or related communication strategies. However, they did not substantively integrate climate-related health impacts as an Exposure within the clinical encounter. Indeed, no report in the full-text assessed corpus met the complete P + E + O configuration. In other words, none simultaneously addressed primary healthcare physicians and culturally diverse adult patients, climate-related health impacts recognised or addressed in clinical communication, and intercultural dialogue strategies relevant to adaptation, community resilience, and the doctor–patient relationship. The final evidence base, therefore, did not correspond to an integrated climate-health and intercultural primary-care corpus, but to the strongest available partial intersection, centred on Population + Outcome.

The remaining categories reinforce this interpretation. **P -** only records addressed healthcare, general practice, health professionals or service users without sufficient connection to climate-related exposure or intercultural dialogue. **E -** only records addressed climate-related health impacts, ecological vulnerability or environmental health concerns without substantive engagement with primary healthcare doctor–patient interaction or intercultural communication. **O -** only records focused on intercultural communication, cultural competence, cultural safety, equity-oriented care or communication barriers, but without an eligible primary healthcare physician–adult patient population and without a substantive climate-related exposure. These findings indicate that the gap lies in integrating the three axes, rather than in the absence of literature on each axis separately.

### 3.3 Corpus profile and analytical usability

The curated bibliometric corpus comprised 745 records, which were retained for the main analyses. The corpus was predominantly scientific literature (*n* = 703; 94.4%), while grey literature comprised 42 records (5.6%). Journal articles were the dominant document type (*n* = 654; 87.8%), followed by theses, dissertations, reports and technical documents (*n* = 39; 5.2%), books and book chapters (*n* = 8; 1.1%) and other document types (*n* = 44; 5.9%). The source-of-origin profile shows that most records entered the curated corpus via the main international bibliographic databases. Scopus, Web of Science and PubMed together accounted for 480 records (64.4%). SciELO and RCAAP contributed 183 records (24.6%), and Google Scholar and other complementary sources accounted for 82 records (11.0%). These categories refer to the database, repository, or search route through which each record entered the corpus, rather than the journal, publisher, or publication venue.

English-language publications dominated the corpus (*n* = 570; 76.5%), followed by Portuguese-language records (*n* = 126; 16.9%). Spanish- and other-language records accounted for 49 documents (6.6%). This distribution reflects both the multilingual search strategy and the continuing predominance of English-language material in the indexed literature retained for analysis. Manual curation further improved the corpus's analytical usability. After curation, all records contained title, author information, year, abstract, publication venue or source, and keywords. DOI availability was high (*n* = 648; 87.0%). In total, 174 records (23.4%) required completion or correction of at least one core field during curation. Keywords were available for all 745 records, allowing the full corpus to be included in keyword co-occurrence analysis. The geographical context was identifiable in 636 records (85.4%). Fifty-one records (6.8%) were excluded from at least one metadata-dependent analysis because the required fields were incomplete, unidentifiable or inapplicable.

### 3.4 Temporal evolution, geographical focus, publication venues and citation visibility

Annual publication output expanded gradually but irregularly across the study period (Figure 2a). Output was relatively modest in the early years, increased after 2007–2012, fluctuated around a marked peak in 2016 and became more consistently higher from 2018 onwards. The strongest growth occurred in the most recent period, particularly between 2022 and 2025, with the highest annual output observed in 2025. The apparent decline in 2026 should be interpreted cautiously because the search covered only records available up to 31 March 2026.

**Figure 2:**
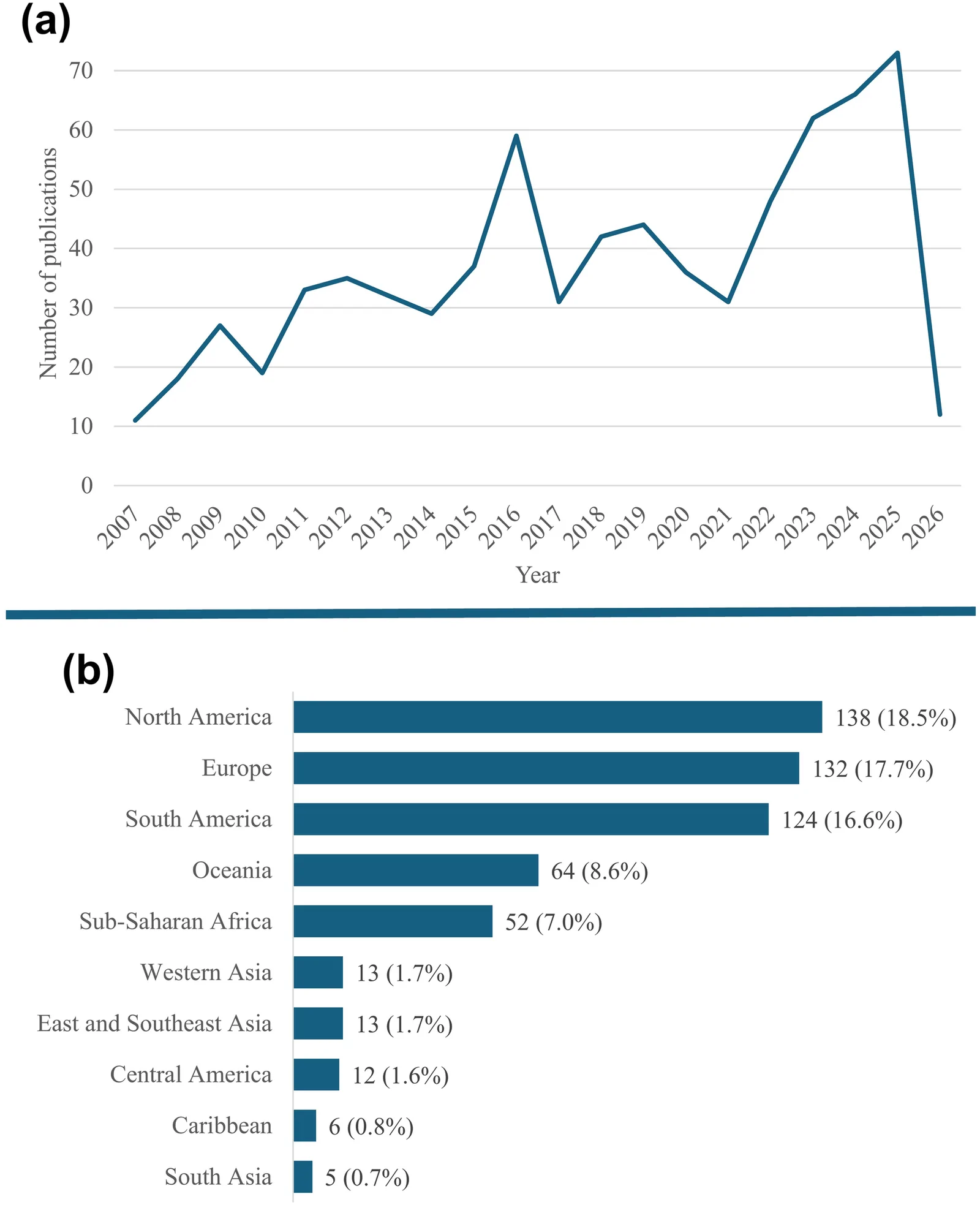
Temporal evolution and geographical focus of the curated bibliometric corpus. Panel **(a)** Data for 2026 cover publications indexed or retrieved up to 31 March 2026 only; therefore, the 2026 count represents a partial year and should not be interpreted as evidence of a decline in publication output. Panel **(b)** Geographical focus of study contexts identified in the corpus.

The geographical profile of study contexts was unevenly distributed (Figure 2b). North America accounted for the largest share of study contexts (*n* = 138; 18.5%), followed by Europe (*n* = 132; 17.7%) and South America (*n* = 124; 16.6%). Oceania accounted for 64 records (8.6%) and Sub-Saharan Africa for 52 records (7.0%). Western Asia, East and Southeast Asia, Central America, the Caribbean, and South Asia had smaller proportions. A further 109 records (14.6%) did not specify a clear country or regional study context in the methods or main text, and 77 records (10.3%) involved multiple countries.

At the country level, the United States of America was the most frequent study context (*n* = 98), followed by Brazil (*n* = 93), Australia (*n* = 59), Canada (*n* = 40) and Portugal (*n* = 30). Other recurrent contexts included South Africa and the United Kingdom (*n* = 21 each), Spain (*n* = 18), the Netherlands (*n* = 12), and Colombia and Mexico (*n* = 11 each). This pattern indicates broad but uneven geographical coverage, with stronger representation from North America, Europe, South America and Oceania, and comparatively limited representation from many Asian, Caribbean and African contexts beyond South Africa. These figures refer to the study context, not to author affiliation, institutional productivity, or country of publication.

Publication output spanned numerous venues, with no single journal or source dominating the corpus. The most frequent venue was the African Journal of Primary Health Care & Family Medicine (*n* = 19; 2.6%), followed by Patient Education and Counseling (*n* = 17; 2.3%). Interestingly, the most frequent publication venue was African, despite Sub-Saharan Africa accounting for a relatively modest proportion of study contexts. This highlights the distinction between publication venues and geographical study settings and suggests that some journals may serve as important interdisciplinary outlets for research on primary care, equity and sociocultural diversity beyond their immediate regional focus. The remaining leading venues each accounted for small proportions of the corpus, generally between 0.8% and 1.6%. Together, the top 20 publication venues represented 190 records, corresponding to approximately one quarter of the corpus.

The profile of leading venues reinforces the corpus's interdisciplinary character. Journals associated with primary healthcare, patient communication, public health, health services research, collective health, migrant and minority health, and general medicine all appeared among the most recurrent sources. The presence of Latin American and Ibero-American journals, including *Ciência & Saúde Coletiva, Revista Panamericana de Salud Pública, Physis: Revista de Saúde Coletiva, Saúde e Sociedade, and Interface—Comunicação, Saúde, Educação*, further indicates the corpus's multilingual and geographically plural character.

The citation profile revealed a visible intellectual layer centred on cultural safety, cultural competence, health equity and socially responsive clinical practice (Table 3). The most globally visible documents addressed cultural safety, implicit racial and ethnic bias among healthcare providers, social determinants of health in clinical practice, Hispanic/Latino health, and health professionals’ views on climate change and health. A second visible group focused on migrant and minority health, culturally and linguistically diverse populations, access to care and medication adherence.

**Table 3:** Most globally visible documents in the curated bibliometric corpus.

Rank	Year	Title	Global citations	Dominant thematic domain
1	2019	Why cultural safety rather than cultural competency is required to achieve health equity: A literature review and recommended definition (40)	963	Cultural safety, cultural competence and health equity
2	2018	A decade of studying implicit racial/ethnic bias in healthcare providers using the implicit association test (41)	664	Implicit bias, racism and healthcare disparities
3	2016	Taking action on the social determinants of health in clinical practice: A framework for health professionals (42)	565	Social determinants and equity-oriented clinical practice
4	2016	Hispanic health in the USA: A scoping review of the literature (43)	434	Hispanic/Latino health, social determinants and access to care
5	2021	Views of health professionals on climate change and health: a multinational survey study (35)	433	Climate change, health professionals and global health
6	2007	Cultural competence in mental health care: A review of model evaluations (44)	325	Cultural competence and mental health services
7	2011	Does cultural competency training of health professionals improve patient outcomes? A systematic review and proposed algorithm for future research (45)	298	Cultural competence training and patient outcomes
8	2022	Access to health services among culturally and linguistically diverse populations in the Australian universal health care system: issues and challenges (46)	274	Access to care for culturally and linguistically diverse populations
9	2011	Good practice in health care for migrants: Views and experiences of care professionals in 16 European countries (47)	264	Migrant health and professional practice
10	2018	Cultural Issues in Medication Adherence: Disparities and Directions (48)	222	Culture, medication adherence and health disparities

Citation counts refer to global citation counts retrieved from Scopus on 9 May 2026. Because citation coverage varies across databases and document types, these records are interpreted as globally visible documents rather than as definitive measures of intellectual influence.

The authors assigned the thematic domain based on the title, abstract, and full text, where necessary.

Climate change appeared less frequently among the most-cited documents globally. The citation profile suggests the corpus's intellectual foundations are rooted primarily in cultural safety, cultural competence, health equity, migrant and minority health, and socially responsive clinical practice. These themes account for most of the globally visible documents and appear to provide a shared conceptual framework across otherwise diverse strands of the literature. The inclusion of a recent multinational survey on health professionals’ views of climate change and health (35) among the ten most-cited records indicates that climate-health has emerged as a visible area of interest within the broader corpus. However, compared with the more established literature on cultural competence, health equity and migrant health, climate-health remains a comparatively newer and less deeply embedded component of the field's citation structure. Overall, the citation profile reflects a research landscape organised around interconnected concerns with equity, diversity, communication and professional practice, rather than around a fully integrated climate-health and intercultural primary-care agenda.

Co-authorship analysis revealed a collaboration structure organised around specialised, only partially connected author groups rather than a single cohesive authorship network (Figure 3). After author-name normalisation, 3,412 authors met the minimum threshold of one document, but only 46 authors formed the largest connected component displayed in the map. This component was structured around several internally cohesive clusters with limited cross-cluster integration. A cluster centred on Sherilee L. Harper appeared on the left side of the map, connected to authors working on climate health, Indigenous health, and related topics, including Ashlee Cunsolo and Gwen K. Healey Akearok. A central group included authors such as Lea Berrang-Ford, Shuaib Lwasa and Didacus Bambaiha Namanya. At the same time, a dense Latin American cluster appeared on the right side of the map and was connected to the wider component primarily through James D. Ford. Overall, the map suggests that collaboration in this corpus was concentrated within specialised teams, with relatively limited cross-thematic or cross-regional bridging.

**Figure 3:**
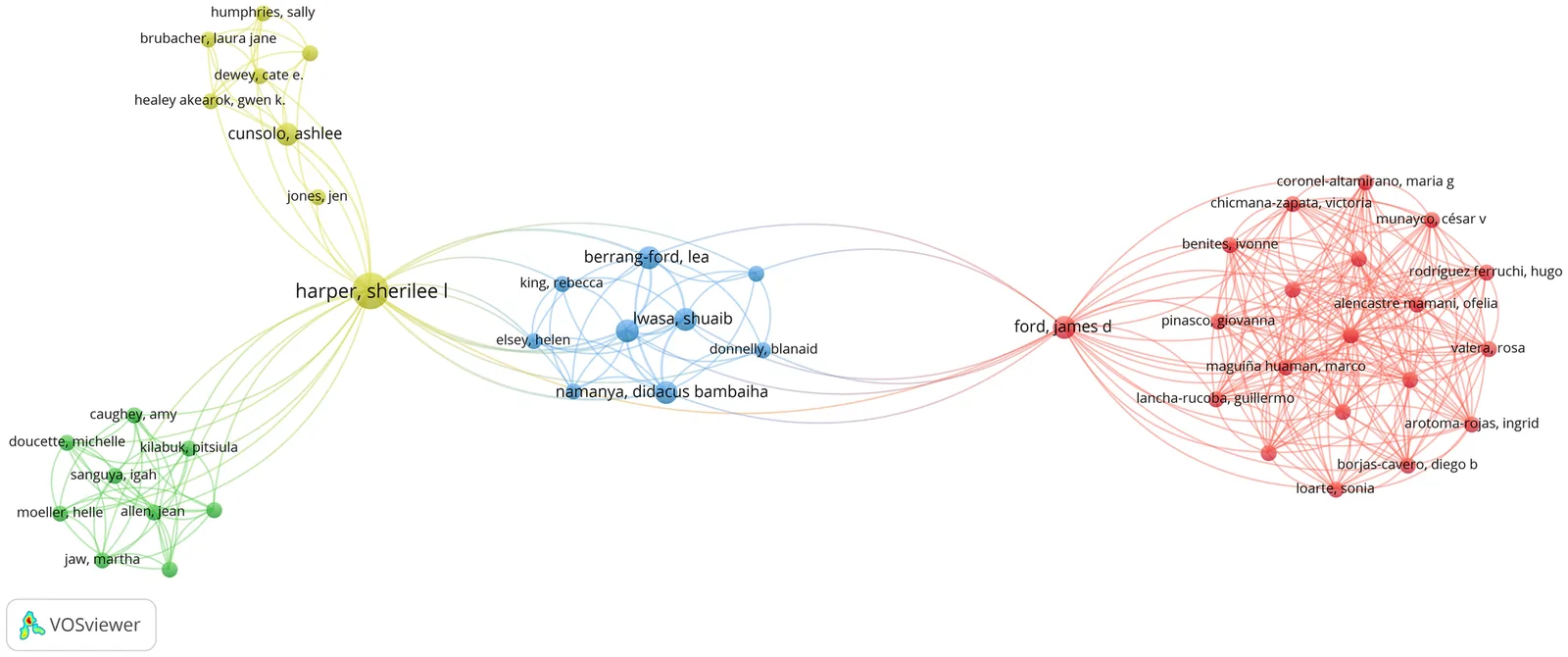
Co-authorship network and temporal overlay of connected authors in the curated bibliometric corpus. The map was generated in VOSviewer using co-authorship analysis at the author level and full counting, based on reference manager files derived from the 745 full-text records assessed. An author-name thesaurus was applied to merge variant author names and remove one group-author label. The minimum number of documents per author was set to one, and the option to ignore documents with a large number of authors was not selected. A total of 3,412 authors met the threshold; because many were not connected, only the largest connected component, comprising 46 authors, was selected for visualisation. Each node represents an author, links represent co-authorship relationships, node size reflects the author's weight in the displayed network, and colours indicate the average publication year according to the VOSviewer overlay scale.

The co-authorship structure reinforces the broader pattern of fragmentation observed throughout the study. Rather than forming a single integrated research community, authors appear to collaborate primarily within specialised thematic and regional networks. The limited connectivity between clusters suggests that knowledge production remains organised around partially overlapping research traditions, including climate-health, Indigenous health, primary healthcare, health equity and intercultural communication. This pattern mirrors the thematic and citation analyses, which likewise indicate convergence around related concerns without the emergence of a fully integrated research field. This interpretation should be considered alongside the fact that only 46 of the 3,412 authors meeting the minimum threshold were included in the largest connected component; the predominance of authors outside this component may partly reflect common patterns of small-team or single-author output in this heterogeneous corpus, such as theses and dissertations, rather than only evidence of thematic fragmentation.

### 3.5 Thematic structure and temporal evolution of the study axes

The keyword co-occurrence map of the 745 full-text-assessed records showed a dense central thematic core linking health, culture, access to health care, cultural competence, primary health care, health care professionals, intercultural communication, communication barriers, doctor–patient communication, migrant populations, and sociocultural diversity (Figure 4). This structure indicates that the corpus's dominant thematic organisation centred on healthcare access, clinical communication, professional practice, cultural competence, and sociocultural diversity.

**Figure 4:**
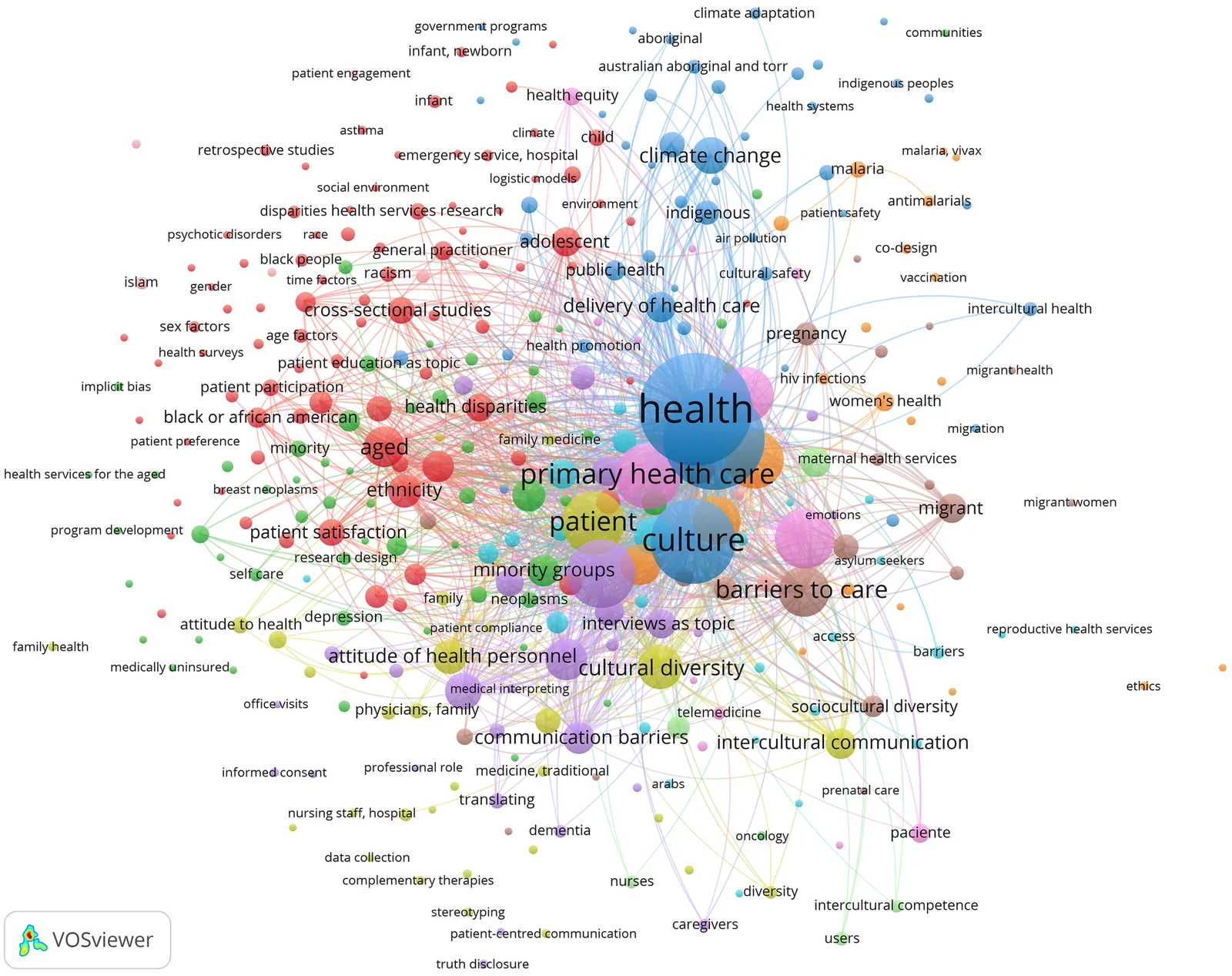
Keyword co-occurrence map of the full-text assessed corpus. The map was generated in VOSviewer using keyword co-occurrence analysis based on the 745 full-text assessed records. Database keywords were used as the unit of analysis. Full counting was applied, with a minimum occurrence threshold of three. Node size reflects keyword occurrence, links indicate co-occurrence relationships, and colours represent thematic clusters. Keyword variants were harmonised through a VOSviewer thesaurus applied only to standardise variants referring to the same analytical term, without broader conceptual recoding. Administrative, source-related and purely locational terms were removed prior to mapping.

Climate-related terms appeared in a more peripheral area of the network. Terms such as climate change, climate adaptation, climate-related health impacts, environmental health, extreme weather, hot temperature, and vector-borne diseases connected to the main network through broader bridging terms, including public health, mental health, Indigenous health, general practice, health equity, and health services. This structure suggests that the corpus was not merely heterogeneous but conceptually fragmented: relevant literature spans the three domains, yet the climate-health dimension remains only partially integrated with the literature on intercultural communication and primary healthcare.

The temporal overlay added a dynamic layer to this interpretation (Figure 5). Central terms such as health, culture, access to health care, cultural competence, primary health care, health care professionals, intercultural communication, doctor–patient communication, migrant populations and sociocultural diversity represented a more established thematic core. By contrast, several climate-health and Indigenous/community-health terms showed more recent average publication years, particularly climate change, climate adaptation, environmental health, hot temperature, vector-borne diseases, Indigenous knowledge, and Indigenous peoples. Their position in the network suggests an emerging but still peripheral thematic axis.

**Figure 5:**
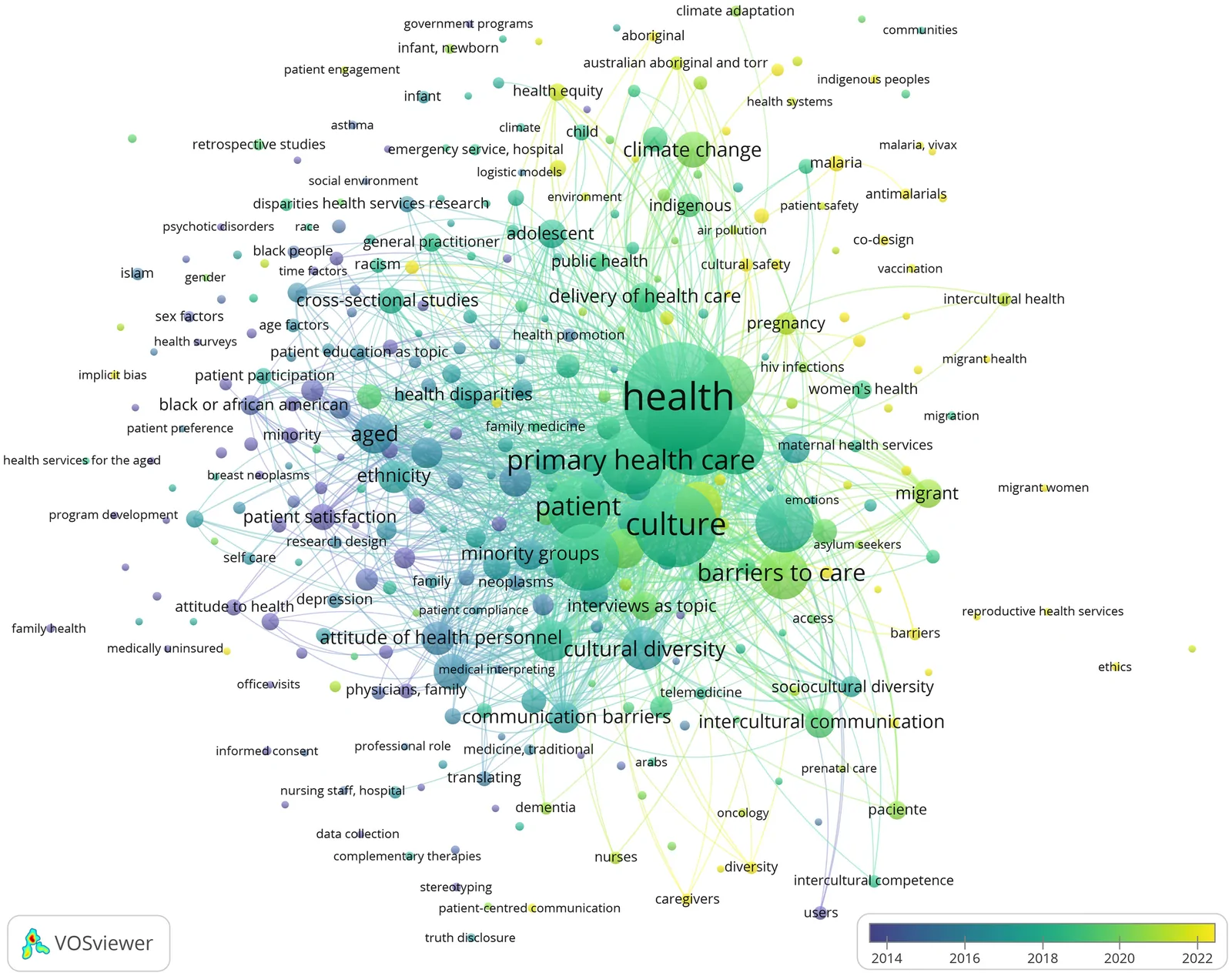
Temporal overlay map of keyword co-occurrence in the full-text assessed corpus. The map was generated in VOSviewer using the same keyword co-occurrence dataset and parameters as Figure 4. Colours indicate the average publication year of keywords according to the VOSviewer overlay scale, allowing visual identification of more established and more recent thematic areas.

Table 4 reports selected thematic hotspots from the keyword co-occurrence map. The terms classified as established central terms—cultural competence, primary health care, access to health care, and communication—showed comparatively high occurrence counts and total link strength, supporting their interpretation as central elements of the thematic structure observed in the map. In contrast, climate-health terms such as climate change, environmental health, climate adaptation, and vector-borne diseases were less frequent, generally more recent, and less structurally embedded.

**Table 4:** Selected thematic hotspots from the keyword co-occurrence map.

Keyword	Cluster	Occurrences	Total link strength	Average publication year	Interpretive category
Cultural competence	5	202	2,043	2018.5	Established central term
Primary health care	9	190	1,664	2018.46	Established central term
Access to health care	7	149	1,444	2018.54	Established central term
Communication	5	79	894	2017.97	Established central term
General practice	5	25	291	2018.16	Primary-care bridging term
Public health	3	19	193	2018.84	Bridging term
Health equity	9	15	160	2021.27	Bridging term
Indigenous health	3	9	96	2020.67	Bridging term
Climate change	3	58	283	2020.05	Climate-health term
Environmental health	1	13	48	2021.46	Climate-health term
Vector-borne diseases	7	11	57	2023.45	Emerging climate-health term
Climate adaptation	3	6	26	2020.33	Climate-health term

Keywords were selected from the final VOSviewer keyword co-occurrence file used to generate Figures 4, 5. Occurrences indicate the number of records in which the term appeared, and total link strength indicates the overall strength of co-occurrence links with other terms in the map. Average publication year refers to the mean publication year of records containing the keyword. The authors assigned interpretive categories to support reading the thematic structure. Established central terms refer to high-occurrence, highly connected keywords within the network's central thematic structure. Bridging terms refer to keywords that connect the central healthcare/intercultural communication core with adjacent thematic areas. Climate-health terms refer to keywords directly related to climate change or climate-sensitive health impacts; emerging climate-health terms refer to climate-health keywords with comparatively recent average publication years. We removed geographical names when they functioned only as location descriptors, because we analysed geographical distribution separately; we retained population, cultural, ethnic, or identity-related terms when analytically relevant to sociocultural diversity, interculturality, and health equity. The map used full counting, a minimum occurrence threshold of three, and included 344 keywords distributed across 11 clusters.

This thematic pattern corresponds to the evidence gap identified through the PEO classification. The absence of a consolidated P + E + O configuration does not mean that the corpus lacks literature on climate change, healthcare or intercultural communication as separate domains. Rather, it indicates that these domains are usually articulated separately or only partially intersect. The strongest and most consolidated axis was P + O, linking primary healthcare, health professionals, patients or service users, cultural competence, intercultural communication, migration, sociocultural diversity, trust and access to care. The Exposure dimension, represented by climate-related health impacts and ecological vulnerability, was visible but comparatively peripheral.

Taken together, the PEO classification and keyword maps show that the field is thematically convergent but not yet analytically integrated. The corpus includes mature work on cultural competence, migrant health, communication barriers, clinical relationships, and access to care, alongside a more recent, less structurally embedded climate-health literature. The main gap lies in the limited articulation between these strands, particularly the limited integration of climate-related health impacts into face-to-face intercultural communication between primary-care professionals and culturally diverse patients.

## 4 Discussion

Within the predefined PEO framework, this systematic bibliometric analysis shows that the literature surrounding primary healthcare, climate-related health impacts and intercultural dialogue is substantial but only partially integrated. The decision to analyse the full-text assessed corpus, rather than only the final qualitative sample, was particularly important for identifying this fragmentation. Had the analysis been restricted to the 64 studies retained for qualitative follow-up, the broader relationships among adjacent literatures would have remained largely invisible. Full-text assessment established that no report met the complete P + E + O configuration, while bibliometric analysis of the 745-record corpus made it possible to identify the thematic and bibliographic pathways that partially connect the three domains. The gap therefore lies not in the absence of research on the three domains separately, but in the absence of a consolidated body of work combining them within the primary-care clinical encounter. The strongest available partial intersection was P + O, comprising studies on primary healthcare, culturally diverse adult patients, health professionals, intercultural communication, cultural competence, cultural safety or equity-oriented practice, but without substantive integration of climate-related health impacts as an Exposure within clinical communication. This pattern was reinforced by the keyword maps: the central thematic core was organised around access to care, communication, cultural competence, primary healthcare, migrant populations and sociocultural diversity, whereas climate-related terms remained more peripheral and were connected mainly through broader terms such as public health, mental health, Indigenous health, general practice, health equity and health services. Overall, the retrieved literature appears thematically convergent but analytically fragmented within the predefined PEO framework.

The study has several methodological strengths. The review followed a registered review process and employed an updated, multilingual, multi-source search strategy spanning biomedical, multidisciplinary, regional and grey literature sources. This broad coverage was particularly important because the topic spans primary healthcare, climate-health, and intercultural dialogue. The search strategy directly targeted the complete P + E + O intersection and, where database functionality allowed, also included the P + E, P + O and E + O combinations. This approach was designed to maximise retrieval of literature linking the three domains or their partial intersections, rather than relying solely on evidence identified through single-domain searches. The pre-specified Population–Exposure–Outcome framework guided eligibility assessment and enabled the review to examine not only publication patterns but also the degree of conceptual integration across the three domains. Manual metadata curation, staged deduplication, keyword harmonisation, descriptive performance analysis, keyword co-occurrence analysis, temporal overlay and co-authorship mapping strengthened the analytical usability of a large and heterogeneous corpus.

The findings should also be read in relation to three adjacent bodies of literature rather than as a direct replication of a previous integrated review. The climate-health literature has established climate change as a public health threat, affecting morbidity, mortality, health systems, and health inequalities (1, 2, 7, 8). This literature has also documented specific implications for Indigenous populations, whose health and subsistence systems are disproportionately affected by climate change and depend on integrating diverse knowledge systems into adaptation responses (36, 37). The literature on culture, language, and healthcare communication has shown that sociocultural background, language, trust, and acceptability shape the doctor–patient relationship and influence equitable access to care (9–11). This includes Portuguese- and Spanish-language literature addressing cultural competence in primary and socio-sanitary care with immigrant and ethnic-minority populations (38, 39). Public health and medical literature have also argued that health professionals and health systems have responsibilities in climate mitigation, adaptation, and risk communication (12–14). The literature closest to this intersection includes studies linking climate change, migration and family medicine (17), climate-related health impacts among culturally and linguistically diverse communities (18), and the broader climate–migration–health nexus (23, 24). However, within the predefined PEO framework, these studies still represent partial rather than complete integration of the three domains. This study does not contradict these bodies of literature; rather, it shows that the points of convergence among them remain weakly consolidated within the field's bibliographic and thematic structure. Previous work has generally addressed climate change and health, intercultural communication in healthcare, or professional responsibility in relation to climate change as distinct or adjacent areas. By contrast, this study examined whether these areas are brought together within the same research space. Within the literature retrieved and classified under the predefined PEO framework, the results suggest that these areas are not yet systematically integrated, indicating that the gap reflects not simply an absence of publications but also how research is organised across disciplines, vocabularies, indexing practices, and partially overlapping collaborative networks. This interpretation is also supported by the publication, citation and collaboration patterns observed in the corpus. Relevant research was dispersed across a wide range of publication venues, with no single journal serving as a dominant focal point for the field. Similarly, the citation profile was centred primarily on cultural safety, health equity, migrant and minority health, and socially responsive clinical practice. At the same time, climate-health appeared as a visible but comparatively less embedded strand. The co-authorship network showed a limited number of connections between specialised author clusters, suggesting that researchers working on related topics often remain organised within partially separate thematic and regional communities. Taken together, these findings indicate that the observed fragmentation is not only conceptual but also bibliographic and collaborative.

One possible explanation for this fragmentation is that the three domains are usually framed through different professional and epistemic traditions. Climate-health research is often organised at the population, environmental, or health-system level. In contrast, intercultural healthcare research tends to focus on communication, access, equity, migration, cultural safety, and the clinical relationship. Primary care sits between these levels, but climate-related risks may still be treated as public health or emergency preparedness issues rather than routine clinical communication. This mirrors observations from adjacent reviews, which similarly report weak integration between the climate-migration and primary-care literatures despite each being individually well developed (23, 24). The practical implication is that culturally responsive climate-health adaptation in primary care cannot be built by adding climate information to clinical practice alone. It also requires attention to how patients understand risk, describe symptoms, interpret professional advice and negotiate care in contexts shaped by language, migration history, social position, housing conditions, occupational exposure, food insecurity, displacement or territorial vulnerability. For clinicians, the findings point to communication practices that connect climate-sensitive health risks with culturally situated experiences of illness and care. For policymakers and primary-care organisations, they suggest linking climate adaptation plans to existing work on health equity, cultural safety, interpreter use, cultural mediation, community engagement, and professional training. Without this integration, climate preparedness and intercultural or equity-oriented care may continue to develop as parallel agendas.

Understanding how primary healthcare, climate-related health impacts, and intercultural dialogue relate to one another is important for advancing culturally responsive climate-health adaptation, yet it remains unclear whether these domains constitute an integrated research field. Drawing on a systematic, multilingual search across six databases and grey literature sources, and a full-text assessment of 745 records, this study provides what is, to our knowledge, the first bibliometric mapping of the intersection of primary healthcare (with a focus on primary-care physicians), climate-related health impacts, and intercultural dialogue. Within the predefined Population–Exposure–Outcome (PEO) framework used to define eligibility, these three domains were not identified as a fully integrated body of research: this pattern was evident at the broader screening stage, where only 801 of the 13,816 screened records (5.8%) progressed to full-text retrieval, and was confirmed in the 745-record full-text corpus, where no report met the complete P + E + O configuration. Although the corpus contains substantial literature on cultural competence, migrant and minority health, clinical communication, access to care, health equity, and climate-health, these strands remain only partially connected. Bibliometric mapping further showed that climate-related health impacts were comparatively peripheral to the more established thematic core centred on primary healthcare, communication, cultural competence, and sociocultural diversity. These findings suggest that culturally responsive climate-health adaptation in primary care requires closer integration of climate preparedness with existing approaches to health equity, cultural safety, and intercultural communication.

This interpretation should also be considered in light of the study's limitations. A single lead author conducted screening and data curation. Although verification procedures and supervisory review improved consistency, they do not replace full dual independent screening. The bibliometric analysis covered the full 745-report corpus, labelled in the PRISMA flow diagram (Figure 1) as the bibliometric corpus analysed in this article; the 64-report P + O subset, labelled there as the reports retained for subsequent qualitative synthesis, is nested within it. Because the 745-report corpus spans the full range of conceptual PEO classifications shown in the diagram—including PEO mismatch and P-only, E-only and O-only records, in addition to the P + O subset—the resulting thematic and collaborative patterns may partly reflect this broader composition and should be interpreted accordingly. In addition, some reports could not be retrieved in full text, and the analyses depended on the completeness and consistency of the available metadata, keywords and citation data, which may underrepresent concepts discussed in the full texts but not reflected in indexed terms. Eligibility was restricted to physicians rather than the wider primary-care workforce; therefore, the findings should not be generalised to nurses, community health workers, or other multidisciplinary primary-care professionals, whose role in culturally responsive climate-health communication merits dedicated investigation.

Future research should shift from adjacent literatures towards direct empirical work at the P + E + O intersection. Studies are needed to examine how primary-care professionals and culturally diverse adult patients recognise, discuss, and respond to climate-related health impacts in real clinical encounters. Qualitative, mixed-methods, and participatory studies could examine how language, trust, cultural meanings, legal status, socioeconomic position, and prior experiences with health services shape communication about heat, air quality, vector-borne diseases, food insecurity, displacement, housing vulnerability, or extreme weather. Intervention studies could assess whether cultural mediation, interpreter-supported climate-health advice, community-linked primary-care models, culturally adapted decision aids, or professional training in climate-sensitive intercultural communication improve understanding, trust, preparedness, and access to care. This research agenda should also extend to geographical contexts underrepresented in the retrieved literature, particularly where climate vulnerability, sociocultural diversity, and barriers to primary care converge. This aligns with prior evidence that primary-care perspectives on climate change remain particularly underexplored in under-resourced settings such as Sub-Saharan Africa (17). The uneven geographical distribution observed in the corpus reinforces this priority, although the present data do not allow conclusions about the causes of regional underrepresentation. The qualitative synthesis from the P + O subset may help identify transferable principles, but it should be understood as a bridge to integrated research rather than a substitute for studies that directly examine climate-related health communication in culturally diverse primary-care settings.

## Data Availability

The raw data supporting the conclusions of this article will be made available by the authors, without undue reservation.
