# Supplementary material for: Primary healthcare, climate-related health impacts and intercultural dialogue: bibliometric evidence of a fragmented research field
Source: Front Health Serv. 2026 Sep 30;6:1909093. doi: 10.3389/frhs.2026.1909093 (PMC13662001; doi:10.3389/frhs.2026.1909093)
Supplement: Supplementary file 2 [file Datasheet2.pdf]

Supplementary File S2: Full Search Strategies and Source-Specific Search Results

Primary healthcare, climate-related health impacts and intercultural dialogue: bibliometric evidence of a fragmented research field

Authors: Nidia Ponte, Fátima Alves, Diogo Guedes Vidal

Journal: Frontiers in Health Services

Article type: Systematic Review

PROSPERO registration: CRD420251038912

OSF registration: 10.17605/OSF.IO/AXYDW

This document is the supplementary file that includes the information about:

- The full search strategies used in the review, including the search strategies for each database and information source, copied and pasted as executed.
- The methods of managing records also included, for each search strategy, the date of the last search, as well as the number of records identified from each database and other information sources.
- For all databases, the search strategy structure was constructed using three conceptual blocks aligned with the PEO framework:
    - Population (Block A): Terms related to primary healthcare providers and patients from diverse sociocultural backgrounds.
    - Exposure (Block B): Terms representing climate change and related environmental health risks.
    - Outcomes (Block C): Terms capturing intercultural dialogue and related constructs (intercultural communication, cultural competence, cross-cultural communication, traditional knowledge, cultural diversity, community resilience) and perceptions of clinicians and patients about climate-related health impacts

The initial formal searches were conducted in June 2025. An updated search was conducted on 1 April 2026 to capture records published between 1 June 2025 and 31 March 2026. The complete search window therefore covered publications from 1 January 2007 to 31 March 2026.

In addition to the database searches reported here, records from the reviewer's pre-existing reference library were added when considered relevant to the review topic. An exploratory search was also conducted in Epistemonikos at an early stage of the review to identify existing evidence syntheses and support the refinement of the search strategy and search terms. These records were aggregated and reported under the category "Other sources". They were considered supplementary to the main systematic search process and were subjected to the same de-duplication, screening and eligibility procedures as the records retrieved from the main bibliographic sources.

The three conceptual blocks were combined with AND, while synonyms within each block were linked with OR.

The core search vocabulary was structured into three conceptual blocks, with database-specific adaptations reported in the exact search strings below:

- **Block A – Population**
  "primary care" OR "general practice" OR "family medicine" OR "primary healthcare" OR "general practitioner" OR "primary health care" OR "health professionals" OR "community health workers" OR "healthcare providers"
  AND
  "patient" OR "healthcare service users" OR "immigrant" OR "migrant" OR "ethnic minorities" OR "diverse populations" OR "minority groups"
- **Block B – Exposure**
  "climate change" OR "global warming" OR "extreme weather" OR "heat stress" OR "air pollution" OR "vector-borne diseases" OR "environmental risk" OR "climate-sensitive health" OR "climate-related health risks" OR "environmental change" OR "climate-sensitive health outcomes" OR "temperature-related diseases"
- **Block C – Outcomes**
  "intercultural communication" OR "intercultural dialogue" OR "cultural competence" OR "cross-cultural communication" OR "cross-cultural dialogue" OR "cross-cultural" OR "intercultural health" OR "traditional knowledge" OR "indigenous knowledge" OR "cultural diversity" OR "community resilience"

The term “user” was retained in the Scopus and Web of Science strings as the free-text term recorded in the executed searches. It was used as a broad synonym for health service users and was interpreted during screening in relation to the review’s eligibility criteria.

In each database, relevant document types were selected explicitly during the search process to ensure alignment with the aims of the review. The following publication types were included: journal articles (original research and reviews), book chapters, books, conference papers, and academic theses or dissertations. The term 'reviews' refers to systematic reviews, scoping reviews, integrative reviews, and theoretically grounded narrative or conceptual reviews, provided they present a straightforward methodological approach or offer a significant contribution to the study’s conceptual framework. Editorials, letters, comments, notes, short surveys, abstracts, and errata were excluded—either manually or by applying document type filters available within each database interface.

Database Search Report: Scopus

Database: Scopus (Elsevier)

Date of search execution: 19 June 2025

Timeframe applied: 01.01.2007 to 31.05.2025

Document types included: Article, Review, Book chapter, conference paper, book, conference review

Search fields: Title, Abstract, Keywords

Language restrictions: English, Portuguese, and Spanish (as permitted by the platform interface)

Search query entered before applying interface filters:

Block A+B+C = PEO:

TITLE-ABS-KEY (
("primary care" OR "general practice" OR "family medicine" OR "primary healthcare" OR "general practitioner" OR "primary health care" OR "health professionals" OR "community health workers" OR "healthcare providers") AND ("patient" OR "user" OR "immigrant" OR "migrant" OR "ethnic minorities" OR "diverse populations" OR "minority groups") AND ("climate change" OR "global warming" OR "extreme weather" OR "heat stress" OR "air pollution" OR "vector-borne diseases" OR "environmental risk" OR "climate-sensitive health" OR "climate-related health risks" OR "environmental change" OR "climate-sensitive health outcomes" OR "temperature-related diseases") AND ("intercultural communication" OR "intercultural dialogue" OR "cultural competence" OR "cross-cultural communication" OR "cross-cultural dialogue" OR "cross-cultural" OR "intercultural health" OR "traditional knowledge" OR "indigenous knowledge" OR "cultural diversity" OR "community resilience"))

Block A +B = PE:

TITLE-ABS-KEY (("primary care" OR "general practice" OR "family medicine" OR "primary healthcare" OR "general practitioner" OR "primary health care" OR "health professionals" OR "community health workers" OR "healthcare providers") AND ("patient" OR "user" OR "immigrant" OR "migrant" OR "ethnic minorities" OR "diverse populations" OR "minority groups") AND ("climate change" OR "global warming" OR "extreme weather" OR "heat stress" OR "air pollution" OR "vector-borne diseases" OR "environmental risk" OR "climate-sensitive health" OR "climate-related health risks" OR "environmental change" OR "climate-sensitive health outcomes" OR "temperature-related diseases"))

Block A+C = PO

TITLE-ABS-KEY (("primary care" OR "general practice" OR "family medicine" OR "primary healthcare" OR "general practitioner" OR "primary health care" OR "health professionals" OR "community health workers" OR "healthcare providers") AND ("patient" OR "user" OR "immigrant" OR "migrant" OR "ethnic minorities" OR "diverse populations" OR "minority groups") AND ("intercultural communication" OR "intercultural dialogue" OR "cultural competence" OR

"cross-cultural communication" OR "cross-cultural dialogue" OR "cross-cultural" OR "intercultural health" OR "traditional knowledge" OR "indigenous knowledge" OR "cultural diversity" OR "community resilience"))

Block B+C = EO

TITLE-ABS-KEY (("climate change" OR "global warming" OR "extreme weather" OR "heat stress" OR "air pollution" OR "vector-borne diseases" OR "environmental risk" OR "climate-sensitive health" OR "climate-related health risks" OR "environmental change" OR "climate-sensitive health outcomes" OR "temperature-related diseases") AND ("intercultural communication" OR "intercultural dialogue" OR "cultural competence" OR "cross-cultural communication" OR "cross-cultural dialogue" OR "cross-cultural" OR "intercultural health" OR "traditional knowledge" OR "indigenous knowledge" OR "cultural diversity" OR "community resilience"))

Search Combinations and Results

Scopus	
**Blocks**	**Records retrieved**
Block A + B + C	9
Block A + B	702
Block A + C	2330
Block B + C	4619
**Total**	**7 660**

Database Search Report: Web of Science

Database: Web of Science Core Collection

Date of search execution: 19 June 2025

Timeframe applied: 01.01.2007 to 31.05.2025

Document types included: Article or Other or Review Article or Dissertation Thesis or Early Access or Book

Search fields: Topic (Title, Abstract, Author Keywords, Keywords Plus)

Language restrictions: English, Portuguese, and Spanish

Search query entered before applying interface filters:

Block A+B+C = PEO:

TS=(("primary care" OR "general practice" OR "family medicine" OR "primary healthcare" OR "general practitioner" OR "primary health care" OR "health professionals" OR "community health workers" OR "healthcare providers")
AND ("patient" OR "user" OR "immigrant" OR "migrant" OR "ethnic minorities" OR "diverse populations" OR "minority groups") AND ("climate change" OR "global warming" OR "extreme weather" OR "heat stress" OR "air pollution" OR "vector-borne diseases" OR "environmental risk" OR "climate-sensitive health" OR "climate-related health risks" OR "environmental change" OR "climate-sensitive health outcomes" OR "temperature-related diseases") AND ("intercultural communication" OR "intercultural dialogue" OR "cultural competence" OR "cross-cultural communication" OR "cross-cultural dialogue" OR "cross-cultural" OR "intercultural health" OR "traditional knowledge" OR "indigenous knowledge" OR "cultural diversity" OR "community resilience"))

Block A +B = PE:

TS=(("primary care" OR "general practice" OR "family medicine" OR "primary healthcare" OR "general practitioner" OR "primary health care" OR "health professionals" OR "community health workers" OR "healthcare providers")
AND ("patient" OR "user" OR "immigrant" OR "migrant" OR "ethnic minorities" OR "diverse populations" OR "minority groups")
AND ("climate change" OR "global warming" OR "extreme weather" OR "heat stress" OR "air pollution" OR "vector-borne diseases" OR "environmental risk" OR "climate-sensitive health" OR "climate-related health risks" OR "environmental change" OR "climate-sensitive health outcomes" OR "temperature-related diseases"))

Block A+C = PO:

TS=(("primary care" OR "general practice" OR "family medicine" OR "primary healthcare" OR "general practitioner" OR "primary health care" OR "health professionals" OR "community health workers" OR "healthcare providers") AND ("patient" OR "user" OR "immigrant" OR

"migrant" OR "ethnic minorities" OR "diverse populations" OR "minority groups") AND ("intercultural communication" OR "intercultural dialogue" OR "cultural competence" OR "cross-cultural communication" OR "cross-cultural dialogue" OR "cross-cultural" OR "intercultural health" OR "traditional knowledge" OR "indigenous knowledge" OR "cultural diversity" OR "community resilience"))

Block B+C = EO:

TS=(("climate change" OR "global warming" OR "extreme weather" OR "heat stress" OR "air pollution" OR "vector-borne diseases" OR "environmental risk" OR "climate-sensitive health" OR "climate-related health risks" OR "environmental change" OR "climate-sensitive health outcomes" OR "temperature-related diseases") AND ("intercultural communication" OR "intercultural dialogue" OR "cultural competence" OR "cross-cultural communication" OR "cross-cultural dialogue" OR "cross-cultural" OR "intercultural health" OR "traditional knowledge" OR "indigenous knowledge" OR "cultural diversity" OR "community resilience"))

Search Combinations and Results

**Web of Science**	
**Blocks**	**Records retrieved**
Block A + B + C	6
Block A + B	315
Block A + C	1595
Block B + C	4353
**Total**	**6 269**

Database Search Report: PubMed

Database: PubMed (National Library of Medicine)

Date of search execution: 19 June 2025

Timeframe applied: 01.01.2007 to 31.05.2025

Search fields: Title and Abstract ([tiab])

Language restrictions: No language filters were available via the platform interface

Document types included: Books and Documents, Case Reports, Classical Article, Congress, English Abstract, Interview, Introductory Journal Article, Lecture, Legislation, Meta-Analysis, Newspaper Article, Observational Study, Review, Scientific Integrity Review, Scoping Review, Systematic Review, Technical Report

Search query entered before applying interface filters:

Block A+B+C = PEO:

("primary care"[tiab] OR "general practice"[tiab] OR "family medicine"[tiab] OR "primary healthcare"[tiab] OR "general practitioner"[tiab] OR "primary health care"[tiab] OR "health professionals"[tiab] OR "community health workers"[tiab] OR "healthcare providers"[tiab]) AND ("patient"[tiab] OR "healthcare service users"[tiab] OR "immigrant"[tiab] OR "migrant"[tiab] OR "ethnic minorities"[tiab] OR "diverse populations"[tiab] OR "minority groups"[tiab]) AND ("climate change"[tiab] OR "global warming"[tiab] OR "extreme weather"[tiab] OR "heat stress"[tiab] OR "air pollution"[tiab] OR "vector-borne diseases"[tiab] OR "environmental risk"[tiab] OR "climate-sensitive health"[tiab] OR "climate-related health risks"[tiab] OR "environmental change"[tiab] OR "climate-sensitive health outcomes"[tiab] OR "temperature-related diseases"[tiab]) AND ("intercultural communication"[tiab] OR "intercultural dialogue"[tiab] OR "cultural competence"[tiab] OR "cross-cultural communication"[tiab] OR "cross-cultural dialogue"[tiab] OR "cross-cultural"[tiab] OR "intercultural health"[tiab] OR "traditional knowledge"[tiab] OR "indigenous knowledge"[tiab] OR "cultural diversity"[tiab] OR "community resilience"[tiab])

Block A +B = PE:

("primary care"[tiab] OR "general practice"[tiab] OR "family medicine"[tiab] OR "primary healthcare"[tiab] OR "general practitioner"[tiab] OR "primary health care"[tiab] OR "health professionals"[tiab] OR "community health workers"[tiab] OR "healthcare providers"[tiab]) AND ("patient"[tiab] OR "healthcare service users"[tiab] OR "immigrant"[tiab] OR "migrant"[tiab] OR "ethnic minorities"[tiab] OR "diverse populations"[tiab] OR "minority groups"[tiab]) AND ("climate change"[tiab] OR "global warming"[tiab] OR "extreme weather"[tiab] OR "heat stress"[tiab] OR "air pollution"[tiab] OR "vector-borne diseases"[tiab] OR "environmental risk"[tiab] OR "climate-sensitive health"[tiab] OR "climate-related health risks"[tiab] OR "environmental change"[tiab] OR "climate-sensitive health outcomes"[tiab] OR "temperature-related diseases"[tiab])

Block A+C = PO

("primary care"[tiab] OR "general practice"[tiab] OR "family medicine"[tiab] OR "primary healthcare"[tiab] OR "general practitioner"[tiab] OR "primary health care"[tiab] OR "health professionals"[tiab] OR "community health workers"[tiab] OR "healthcare providers"[tiab]) AND ("patient"[tiab] OR "healthcare service users"[tiab] OR "immigrant"[tiab] OR "migrant"[tiab] OR "ethnic minorities"[tiab] OR "diverse populations"[tiab] OR "minority groups"[tiab]) AND ("intercultural communication"[tiab] OR "intercultural dialogue"[tiab] OR "cultural competence"[tiab] OR "cross-cultural communication"[tiab] OR "cross-cultural dialogue"[tiab] OR "cross-cultural"[tiab] OR "intercultural health"[tiab] OR "traditional knowledge"[tiab] OR "indigenous knowledge"[tiab] OR "cultural diversity"[tiab] OR "community resilience"[tiab])

Block B+C = EO

("climate change"[tiab] OR "global warming"[tiab] OR "extreme weather"[tiab] OR "heat stress"[tiab] OR "air pollution"[tiab] OR "vector-borne diseases"[tiab] OR "environmental risk"[tiab] OR "climate-sensitive health"[tiab] OR "climate-related health risks"[tiab] OR "environmental change"[tiab] OR "climate-sensitive health outcomes"[tiab] OR "temperature-related diseases"[tiab]) AND ("intercultural communication"[tiab] OR "intercultural dialogue"[tiab] OR "cultural competence"[tiab] OR "cross-cultural communication"[tiab] OR "cross-cultural dialogue"[tiab] OR "cross-cultural"[tiab] OR "intercultural health"[tiab] OR "traditional knowledge"[tiab] OR "indigenous knowledge"[tiab] OR "cultural diversity"[tiab] OR "community resilience"[tiab])

Search Combinations and Results

**Blocks**	**Docs. PubMed**
Block A + B + C	3
Block A + B	31
Block A + C	98
Block B + C	104
**Total**	**236**

Database Search Report: SciELO

Database: SciELO – Scientific Electronic Library Online

Date of search execution: 20 June 2025

Timeframe applied: 01.01.2007 to 31.05.2025

Search fields: All indexes

Language filters: Portuguese and English

Document types included: Selection to be conducted manually during screening phase

Notes on database constraints: Advanced Boolean nesting and field-specific search were not fully supported

Search Strategy Adaptation

In SciELO, the search strategy had to be adapted to the platform's structural limitations, particularly its restricted Boolean functionality and inconsistent indexing across languages. Initial attempts to run full combined queries (A + B + C), even using the same block combinations successfully applied in other databases, resulted in zero returns—despite adhering to the recommended syntax. To address this, a sequential block-by-block approach was implemented, whereby each conceptual dimension (Population, Exposure, Outcomes) was searched independently and in two languages: English and Portuguese.

Although SciELO supports content in English, Portuguese, and Spanish, most records—regardless of abstract language—are published and indexed primarily in Portuguese. Consequently, the Spanish-language search was not conducted separately, as Spanish articles are systematically retrieved through Portuguese queries due to SciELO's editorial practices. Document type and thematic relevance will be evaluated manually during the screening stage, given the platform's limited filtering capabilities.

Block A ENG:

primary care OR general practice OR family medicine OR primary healthcare OR general practitioner OR primary health care OR health professionals OR community health workers OR healthcare providers

AND

patient OR healthcare service users OR immigrant OR migrant OR ethnic minorities OR diverse populations OR minority groups

Block A PT:

saúde primária OR medicina geral OR medicina familiar OR cuidados de saúde primários OR médico de medicina geral e familiar OR profissionais de saúde OR agentes comunitários de saúde OR prestadores de cuidados de saúde

AND

paciente OR utentes OR imigrante OR migrante OR minorias étnicas OR populações diversas OR grupos minoritários

Block B ENG:

climate change OR global warming OR extreme weather OR heat stress OR air pollution OR vector-borne diseases OR environmental risk OR climate-sensitive health OR climate-related health risks OR environmental change OR climate-sensitive health outcomes OR temperature-related diseases

Block B PT:

alterações climáticas OR mudanças climáticas OR aquecimento global OR fenómenos meteorológicos extremos OR stresse térmico OR poluição atmosférica OR doenças transmitidas por vetores OR risco ambiental OR saúde sensível ao clima OR riscos para a saúde relacionados com o clima OR alterações ambientais OR resultados de saúde sensíveis ao clima OR doenças relacionadas com a temperatura

Block C ENG:

intercultural communication OR cultural competence OR intercultural dialogue OR cross-cultural communication OR cross-cultural dialogue OR cross-cultural OR intercultural health OR traditional knowledge OR indigenous knowledge OR cultural diversity OR community resilience

Block C PT:

comunicação intercultural OR competência cultural OR diálogo intercultural OR comunicação transcultural OR diálogo transcultural OR transcultural OR saúde intercultural OR conhecimento tradicional OR saberes indígenas OR diversidade cultural OR resiliência comunitária

Search Combinations and Results

**Blocks**	**SciELO**	
	**Collect**	**Total Collect**
A-ENG	1610	1883
A- PT	273	
B - ENG	143	143
B-PT	0	
C- ENG	2	120
C-PT	118	
**Total**		**2 146**

Database Search Report: RCAAP

Database: RCAAP (Repositórios Científicos de Acesso Aberto de Portugal)

Date of search execution: 20, 21 and 23 June 2025

Timeframe applied: 01.01.2007 to 31.05.2025

Search fields: Title and Abstract

Language restrictions: Portuguese only

Document types included: Selection to be conducted manually during screening phase

Due to technical constraints of the RCAAP platform—such as limited Boolean handling, absence of query export tools, and incompatibility with advanced reference managers—searches were conducted manually using simplified combinations of keywords in Portuguese only. Queries were entered into the search field, which proved to be the most effective for capturing thematically indexed documents. A total of 286 distinct searches were conducted. These queries combined terms from the PEO-based conceptual blocks as follows:

- Block A (Population) terms were paired with terms from Block B (Exposure) or Block C (Outcomes), using simplified Boolean syntax (e.g., medicina familiar OR profissionais de saúde AND alterações climáticas).
- Some searches also involved combinations of Block B and C directly (e.g., alterações climáticas AND comunicação intercultural).

In contrast to other platforms, all retrieved documents were manually screened. Given the generally low volume of results per query, often fewer than 10 and only exceptionally above 50, each record's title and abstract were reviewed immediately to determine whether they aligned with the inclusion criteria. A technical limitation also influenced this decision: it was not possible to capture documents directly within RCAAP. Instead, each item had to be accessed at its source repository, where Zotero could be used to record the reference. Relevant documents were then saved for subsequent analysis within the Rayyan platform. In total, 700 documents were reviewed, and **119 were selected** for further analysis within Rayyan.

Search Combinations and Results

**Blocks**	**Queries**	**Doc. RCAAP**
A + B	77	11
A + C	77	87
B + C	132	21
**Total**	**286**	**119**

Database Search Report: Google Scholar

Database: Google Scholar

Date of search execution: 24-06-2025

Timeframe applied: 01.01.2007 to 31.05.2025

Search fields: Full-text (platform-defined default)

Language limitations: No automatic filters were applied.

Search strategy:

Given the platform's broad indexing scope and limited filtering capabilities, a tailored strategy was implemented for Google Scholar. A comprehensive query combining all three conceptual blocks (Population, Exposure, Outcome) was constructed and adapted to each of the review's working languages (English, Portuguese, and Spanish). The query employed Boolean logic, utilising "OR" within blocks and "AND" between blocks and was executed through the platform's advanced search interface.

Each language version returned a substantial number of results: 17,800 in English, 1,360 in Portuguese, and 4,110 in Spanish. To ensure methodological feasibility and maintain relevance, only the first 100 results per language (sorted by relevance) were reviewed. Titles and short descriptions were read to assess alignment with the PEO framework. Documents considered potentially eligible were saved using Zotero and imported into Rayyan for further screening.

Due to technical limitations during the Zotero capture process—such as broken links or incomplete metadata—only a portion of the top 100 results could be successfully saved in each language: 80 documents in English, 55 in Portuguese, and 17 in Spanish, totalling 152 records. These were subsequently integrated into the systematic screening process.

Although preliminary relevance screening was performed based on titles and descriptions at the point of collection, a complete eligibility assessment was reserved for the central review phase. The decision to limit inclusion to the first 100 results per language is consistent with established practices in systematic reviews that use Google Scholar, where search precision is inherently limited, and document types cannot be filtered directly. This approach aimed to capture high-ranking and thematically pertinent documents that may not be indexed in traditional bibliographic databases, thus enhancing the inclusivity and scope of the review.

Search query entered before applying interface filters:

English:

Field: "with all of the words"

primary care OR general practice OR family medicine OR primary healthcare OR general practitioner OR primary health care OR health professionals OR community health workers OR healthcare providers AND patient OR healthcare service users OR immigrant OR migrant OR ethnic minorities OR diverse populations OR minority groups

Field: "with at least one of the words":

climate change OR global warming OR extreme weather OR heat stress OR air pollution OR vector-borne diseases OR environmental risk OR climate-sensitive health OR climate-related health risks OR environmental change OR climate-sensitive health outcomes OR temperature-related diseases AND intercultural communication OR cultural competence OR intercultural dialogue OR cross-cultural communication OR cross-cultural dialogue OR cross-cultural OR intercultural health OR traditional knowledge OR indigenous knowledge OR cultural diversity OR community resilience

Portuguese:

Field: "with all of the words":

saúde primária OR medicina geral OR medicina familiar OR cuidados de saúde primários OR médico de medicina geral e familiar OR profissionais de saúde OR agentes comunitários de saúde OR prestadores de cuidados de saúde AND paciente OR utentes OR imigrante OR migrante OR minorias étnicas OR populações diversas OR grupos minoritários

Field: "with at least one of the words":

alterações climáticas OR mudanças climáticas OR aquecimento global OR fenómenos meteorológicos extremos OR stresse térmico OR poluição atmosférica OR doenças transmitidas por vetores OR risco ambiental OR saúde sensível ao clima OR riscos para a saúde relacionados com o clima OR alterações ambientais OR resultados de saúde sensíveis ao clima OR doenças relacionadas com a temperatura AND comunicação intercultural OR competência cultural OR diálogo intercultural OR comunicação transcultural OR diálogo transcultural OR transcultural OR saúde intercultural OR conhecimento tradicional OR saberes indígenas OR diversidade cultural OR resiliência comunitária

Spanish:

Field: "with all of the words":

primaria de salud OR medicina general OR medicina familiar OR atención primaria de salud OR médico general OR profesionales de la salud OR trabajadores comunitarios de salud OR proveedores de servicios de salud AND paciente OR usuarios de los servicios de salud OR inmigrante OR migrante OR minorías étnicas OR poblaciones diversas OR grupos minoritarios

Field: "with at least one of the words":

cambio climático OR calentamiento global OR fenómenos meteorológicos extremos OR estrés térmico OR contaminación del aire OR enfermedades transmitidas por vectores OR riesgo ambiental OR salud sensible al clima OR riesgos para la salud relacionados con el clima OR cambios ambientales OR desenlaces de salud sensibles al clima OR enfermedades relacionadas con la temperatura AND comunicación intercultural OR competencia cultural OR diálogo intercultural OR comunicación transcultural OR diálogo transcultural OR transcultural OR salud intercultural OR conocimiento tradicional OR saberes indígenas OR diversidad cultural OR resiliencia comunitaria

Search Combinations and Results

Blocks	Google Scholar		
	#Doc	Time	Records retrieved
A + B + C ENG	17800	0,14 sec.	80
A + B + C PT	1360	0,19 sec.	55
A + B + C ES	4110	0,13 sec.	17
**Total**			**152**

Database Search Report: Scopus

Database: Scopus (Elsevier)

Date of search execution: 01 April 2026

Timeframe applied: from 2025-06-01 to 2026-03-31

Document types included: Article, Review, Book chapter, conference paper, book, conference review

Search fields: Title, Abstract, Keywords

Language restrictions: English, Portuguese, and Spanish (as permitted by the platform interface)

Search query entered before applying interface filters:

Block A+B+C = PEO:

TITLE-ABS-KEY (
("primary care" OR "general practice" OR "family medicine" OR "primary healthcare" OR "general practitioner" OR "primary health care" OR "health professionals" OR "community health workers" OR "healthcare providers") AND ("patient" OR "user" OR "immigrant" OR "migrant" OR "ethnic minorities" OR "diverse populations" OR "minority groups") AND ("climate change" OR "global warming" OR "extreme weather" OR "heat stress" OR "air pollution" OR "vector-borne diseases" OR "environmental risk" OR "climate-sensitive health" OR "climate-related health risks" OR "environmental change" OR "climate-sensitive health outcomes" OR "temperature-related diseases") AND ("intercultural communication" OR "intercultural dialogue" OR "cultural competence" OR "cross-cultural communication" OR "cross-cultural dialogue" OR "cross-cultural" OR "intercultural health" OR "traditional knowledge" OR "indigenous knowledge" OR "cultural diversity" OR "community resilience"))

Block A +B = PE:

TITLE-ABS-KEY (("primary care" OR "general practice" OR "family medicine" OR "primary healthcare" OR "general practitioner" OR "primary health care" OR "health professionals" OR "community health workers" OR "healthcare providers") AND ("patient" OR "user" OR "immigrant" OR "migrant" OR "ethnic minorities" OR "diverse populations" OR "minority groups") AND ("climate change" OR "global warming" OR "extreme weather" OR "heat stress" OR "air pollution" OR "vector-borne diseases" OR "environmental risk" OR "climate-sensitive health" OR "climate-related health risks" OR "environmental change" OR "climate-sensitive health outcomes" OR "temperature-related diseases"))

Block A+C = PO

TITLE-ABS-KEY (("primary care" OR "general practice" OR "family medicine" OR "primary healthcare" OR "general practitioner" OR "primary health care" OR "health professionals" OR "community health workers" OR "healthcare providers") AND ("patient" OR "user" OR "immigrant" OR "migrant" OR "ethnic minorities" OR "diverse populations" OR "minority groups") AND ("intercultural communication" OR "intercultural dialogue" OR "cultural competence" OR

"cross-cultural communication" OR "cross-cultural dialogue" OR "cross-cultural" OR "intercultural health" OR "traditional knowledge" OR "indigenous knowledge" OR "cultural diversity" OR "community resilience"))

Block B+C = EO

TITLE-ABS-KEY (("climate change" OR "global warming" OR "extreme weather" OR "heat stress" OR "air pollution" OR "vector-borne diseases" OR "environmental risk" OR "climate-sensitive health" OR "climate-related health risks" OR "environmental change" OR "climate-sensitive health outcomes" OR "temperature-related diseases") AND ("intercultural communication" OR "intercultural dialogue" OR "cultural competence" OR "cross-cultural communication" OR "cross-cultural dialogue" OR "cross-cultural" OR "intercultural health" OR "traditional knowledge" OR "indigenous knowledge" OR "cultural diversity" OR "community resilience"))

Search Combinations and Results

**Scopus**	
**Blocks**	**Records retrieved**
Block A + B + C	6
Block A + B	161
Block A + C	321
Block B + C	284
**Total**	**772**

Database Search Report: Web of Science

Database: Web of Science Core Collection

Date of search execution: 01 April 2026

Timeframe applied: from 2025-06-01 to 2026-03-31

Document types included: Article or Other or Review Article or Dissertation Thesis or Early Access or Unspecified or Abstract or Case Report or Book

Search fields: Topic (Title, Abstract, Author Keywords, Keywords Plus)

Language restrictions: English, Portuguese, and Spanish

Search query entered before applying interface filters:

Block A+B+C = PEO:

TS=(("primary care" OR "general practice" OR "family medicine" OR "primary healthcare" OR "general practitioner" OR "primary health care" OR "health professionals" OR "community health workers" OR "healthcare providers")
AND ("patient" OR "user" OR "immigrant" OR "migrant" OR "ethnic minorities" OR "diverse populations" OR "minority groups") AND ("climate change" OR "global warming" OR "extreme weather" OR "heat stress" OR "air pollution" OR "vector-borne diseases" OR "environmental risk" OR "climate-sensitive health" OR "climate-related health risks" OR "environmental change" OR "climate-sensitive health outcomes" OR "temperature-related diseases") AND ("intercultural communication" OR "intercultural dialogue" OR "cultural competence" OR "cross-cultural communication" OR "cross-cultural dialogue" OR "cross-cultural" OR "intercultural health" OR "traditional knowledge" OR "indigenous knowledge" OR "cultural diversity" OR "community resilience"))

Block A +B = PE:

TS=(("primary care" OR "general practice" OR "family medicine" OR "primary healthcare" OR "general practitioner" OR "primary health care" OR "health professionals" OR "community health workers" OR "healthcare providers")
AND ("patient" OR "user" OR "immigrant" OR "migrant" OR "ethnic minorities" OR "diverse populations" OR "minority groups")
AND ("climate change" OR "global warming" OR "extreme weather" OR "heat stress" OR "air pollution" OR "vector-borne diseases" OR "environmental risk" OR "climate-sensitive health" OR "climate-related health risks" OR "environmental change" OR "climate-sensitive health outcomes" OR "temperature-related diseases"))

Block A+C = PO:

TS=(("primary care" OR "general practice" OR "family medicine" OR "primary healthcare" OR "general practitioner" OR "primary health care" OR "health professionals" OR "community health workers" OR "healthcare providers") AND ("patient" OR "user" OR "immigrant" OR

"migrant" OR "ethnic minorities" OR "diverse populations" OR "minority groups") AND ("intercultural communication" OR "intercultural dialogue" OR "cultural competence" OR "cross-cultural communication" OR "cross-cultural dialogue" OR "cross-cultural" OR "intercultural health" OR "traditional knowledge" OR "indigenous knowledge" OR "cultural diversity" OR "community resilience"))

Block B+C = EO:

TS=(("climate change" OR "global warming" OR "extreme weather" OR "heat stress" OR "air pollution" OR "vector-borne diseases" OR "environmental risk" OR "climate-sensitive health" OR "climate-related health risks" OR "environmental change" OR "climate-sensitive health outcomes" OR "temperature-related diseases") AND ("intercultural communication" OR "intercultural dialogue" OR "cultural competence" OR "cross-cultural communication" OR "cross-cultural dialogue" OR "cross-cultural" OR "intercultural health" OR "traditional knowledge" OR "indigenous knowledge" OR "cultural diversity" OR "community resilience"))

Search Combinations and Results

**Web of Science**	
**Blocks**	**Records retrieved**
Block A + B + C	1
Block A + B	57
Block A + C	134
Block B + C	939
**Total**	**1131**

Database Search Report: PubMed

Database: PubMed (National Library of Medicine)

Date of search execution: 01 April 2026

Timeframe applied: from 2025-06-01 to 2026-03-31

Search fields: Title and Abstract ([tiab])

Language restrictions: No language filters were available via the platform interface

Document types included: Books and Documents, Case Reports, Classical Article, Congress, English Abstract, Interview, Introductory Journal Article, Lecture, Legislation, Meta-Analysis, Newspaper Article, Observational Study, Review, Scientific Integrity Review, Scoping Review, Systematic Review, Technical Report

Search query entered before applying interface filters:

Block A+B+C = PEO:

("primary care"[tiab] OR "general practice"[tiab] OR "family medicine"[tiab] OR "primary healthcare"[tiab] OR "general practitioner"[tiab] OR "primary health care"[tiab] OR "health professionals"[tiab] OR "community health workers"[tiab] OR "healthcare providers"[tiab]) AND ("patient"[tiab] OR "healthcare service users"[tiab] OR "immigrant"[tiab] OR "migrant"[tiab] OR "ethnic minorities"[tiab] OR "diverse populations"[tiab] OR "minority groups"[tiab]) AND ("climate change"[tiab] OR "global warming"[tiab] OR "extreme weather"[tiab] OR "heat stress"[tiab] OR "air pollution"[tiab] OR "vector-borne diseases"[tiab] OR "environmental risk"[tiab] OR "climate-sensitive health"[tiab] OR "climate-related health risks"[tiab] OR "environmental change"[tiab] OR "climate-sensitive health outcomes"[tiab] OR "temperature-related diseases"[tiab]) AND ("intercultural communication"[tiab] OR "intercultural dialogue"[tiab] OR "cultural competence"[tiab] OR "cross-cultural communication"[tiab] OR "cross-cultural dialogue"[tiab] OR "cross-cultural"[tiab] OR "intercultural health"[tiab] OR "traditional knowledge"[tiab] OR "indigenous knowledge"[tiab] OR "cultural diversity"[tiab] OR "community resilience"[tiab])

Block A +B = PE:

("primary care"[tiab] OR "general practice"[tiab] OR "family medicine"[tiab] OR "primary healthcare"[tiab] OR "general practitioner"[tiab] OR "primary health care"[tiab] OR "health professionals"[tiab] OR "community health workers"[tiab] OR "healthcare providers"[tiab]) AND ("patient"[tiab] OR "healthcare service users"[tiab] OR "immigrant"[tiab] OR "migrant"[tiab] OR "ethnic minorities"[tiab] OR "diverse populations"[tiab] OR "minority groups"[tiab]) AND ("climate change"[tiab] OR "global warming"[tiab] OR "extreme weather"[tiab] OR "heat stress"[tiab] OR "air pollution"[tiab] OR "vector-borne diseases"[tiab] OR "environmental risk"[tiab] OR "climate-sensitive health"[tiab] OR "climate-related health risks"[tiab] OR "environmental change"[tiab] OR "climate-sensitive health outcomes"[tiab] OR "temperature-related diseases"[tiab])

Block A+C = PO

("primary care"[tiab] OR "general practice"[tiab] OR "family medicine"[tiab] OR "primary healthcare"[tiab] OR "general practitioner"[tiab] OR "primary health care"[tiab] OR "health professionals"[tiab] OR "community health workers"[tiab] OR "healthcare providers"[tiab]) AND ("patient"[tiab] OR "healthcare service users"[tiab] OR "immigrant"[tiab] OR "migrant"[tiab] OR "ethnic minorities"[tiab] OR "diverse populations"[tiab] OR "minority groups"[tiab]) AND ("intercultural communication"[tiab] OR "intercultural dialogue"[tiab] OR "cultural competence"[tiab] OR "cross-cultural communication"[tiab] OR "cross-cultural dialogue"[tiab] OR "cross-cultural"[tiab] OR "intercultural health"[tiab] OR "traditional knowledge"[tiab] OR "indigenous knowledge"[tiab] OR "cultural diversity"[tiab] OR "community resilience"[tiab])

Block B+C = EO

("climate change"[tiab] OR "global warming"[tiab] OR "extreme weather"[tiab] OR "heat stress"[tiab] OR "air pollution"[tiab] OR "vector-borne diseases"[tiab] OR "environmental risk"[tiab] OR "climate-sensitive health"[tiab] OR "climate-related health risks"[tiab] OR "environmental change"[tiab] OR "climate-sensitive health outcomes"[tiab] OR "temperature-related diseases"[tiab]) AND ("intercultural communication"[tiab] OR "intercultural dialogue"[tiab] OR "cultural competence"[tiab] OR "cross-cultural communication"[tiab] OR "cross-cultural dialogue"[tiab] OR "cross-cultural"[tiab] OR "intercultural health"[tiab] OR "traditional knowledge"[tiab] OR "indigenous knowledge"[tiab] OR "cultural diversity"[tiab] OR "community resilience"[tiab])

Search Combinations and Results

**Blocks**	**Docs. PubMed**
Block A + B + C	0
Block A + B	11
Block A + C	28
Block B + C	28
**Total**	**67**

Database Search Report: SciELO

Database: SciELO – Scientific Electronic Library Online

Date of search execution: 01 April 2026

Timeframe applied: from 2025-06-01 to 2026-03-31

Search fields: All indexes

Language filters: Portuguese and English

Document types included: Selection to be conducted manually during screening phase

Notes on database constraints: Advanced Boolean nesting and field-specific search were not fully supported

Search Strategy Adaptation

In SciELO, the search strategy had to be adapted to the platform's structural limitations, particularly its restricted Boolean functionality and inconsistent indexing across languages. Initial attempts to run full combined queries (A + B + C), even using the same block combinations successfully applied in other databases, resulted in zero returns—despite adhering to the recommended syntax. To address this, a sequential block-by-block approach was implemented, whereby each conceptual dimension (Population, Exposure, Outcomes) was searched independently and in two languages: English and Portuguese.

Although SciELO supports content in English, Portuguese, and Spanish, most records—regardless of abstract language—are published and indexed primarily in Portuguese. Consequently, the Spanish-language search was not conducted separately, as Spanish articles are systematically retrieved through Portuguese queries due to SciELO's editorial practices. Document type and thematic relevance will be evaluated manually during the screening stage, given the platform's limited filtering capabilities.

Block A1 ENG:

primary care OR general practice OR family medicine OR primary healthcare OR general practitioner OR primary health care OR health professionals OR community health workers OR healthcare providers

Block A2 ENG:

patient OR healthcare service users OR immigrant OR migrant OR ethnic minorities OR diverse populations OR minority groups

Block A1 PT:
saúde primária OR medicina geral OR medicina familiar OR cuidados de saúde primários OR médico de medicina geral e familiar OR profissionais de saúde OR agentes comunitários de saúde OR prestadores de cuidados de saúde

Block A2 PT:
paciente OR utentes OR imigrante OR migrante OR minorias étnicas OR populações diversas OR grupos minoritários

Block B ENG:
climate change OR global warming OR extreme weather OR heat stress OR air pollution OR vector-borne diseases OR environmental risk OR climate-sensitive health OR climate-related health risks OR environmental change OR climate-sensitive health outcomes OR temperature-related diseases

Block B PT:
alterações climáticas OR mudanças climáticas OR aquecimento global OR fenómenos meteorológicos extremos OR stresse térmico OR poluição atmosférica OR doenças transmitidas por vetores OR risco ambiental OR saúde sensível ao clima OR riscos para a saúde relacionados com o clima OR alterações ambientais OR resultados de saúde sensíveis ao clima OR doenças relacionadas com a temperatura

Block C ENG:
intercultural communication OR cultural competence OR intercultural dialogue OR cross-cultural communication OR cross-cultural dialogue OR cross-cultural OR intercultural health OR traditional knowledge OR indigenous knowledge OR cultural diversity OR community resilience

Block C PT:
comunicação intercultural OR competência cultural OR diálogo intercultural OR comunicação transcultural OR diálogo transcultural OR transcultural OR saúde intercultural OR conhecimento tradicional OR saberes indígenas OR diversidade cultural OR resiliência comunitária

Search Combinations and Results

Blocks	Collect
A1 + A2 -ENG	82
A1 + A2 PT	19
B - ENG	25
B-PT	0
C- ENG	2
C-PT	18
**Total**	**146**

Database Search Report: RCAAP

Database: RCAAP (Repositórios Científicos de Acesso Aberto de Portugal)

Date of search execution: 01 April 2026

Timeframe applied: from 2025-06-01 to 2026-03-31

Search fields: Title and Abstract

Language restrictions: Portuguese only

Document types included: Selection to be conducted manually during screening phase

Due to technical constraints of the RCAAP platform—such as limited Boolean handling, absence of query export tools, and incompatibility with advanced reference managers—searches were conducted manually using simplified combinations of keywords in Portuguese only. Queries were entered into the search field, which proved to be the most effective for capturing thematically indexed documents. A total of 286 distinct searches were conducted. These queries combined terms from the PEO-based conceptual blocks as follows:

- Block A (Population) terms were paired with terms from Block B (Exposure) or Block C (Outcomes), using simplified Boolean syntax (e.g., medicina familiar OR profissionais de saúde AND alterações climáticas).
- Some searches also involved combinations of Block B and C directly (e.g., alterações climáticas AND comunicação intercultural).

In contrast to other platforms, all retrieved documents were manually screened. Given the generally low volume of results per query, often fewer than 10 and only exceptionally above 50, each record's title and abstract were reviewed immediately to determine whether they aligned with the inclusion criteria. A technical limitation also influenced this decision: it was not possible to capture documents directly within RCAAP. Instead, each item had to be accessed at its source repository, where Zotero could be used to record the reference. Relevant documents were then saved for subsequent analysis within the Rayyan platform.

In total, 68 documents were reviewed, and **11 were selected** for further analysis within Rayyan.

Search Combinations and Results

Blocks	Queries	Doc. RCAAP
A + B	12	3
A + C	22	5
B + C	34	3
**Total**	**68**	**11**

Database Search Report: Google Scholar

Database: Google Scholar

Date of search execution: 01 April 2026

Timeframe applied: from 2025-06-01 to 2026-03-31

Search fields: Full-text (platform-defined default)

Language limitations: No automatic filters were applied.

Search strategy:

Given the platform's broad indexing scope and limited filtering capabilities, a tailored strategy was implemented for Google Scholar. A comprehensive query combining all three conceptual blocks (Population, Exposure, Outcome) was constructed and adapted to each of the review's working languages (English, Portuguese, and Spanish). The query employed Boolean logic, utilising "OR" within blocks and "AND" between blocks and was executed through the platform's advanced search interface.

Each language version returned a substantial number of results: 17,100 in English, 136 in Portuguese, and 277 in Spanish. To ensure methodological feasibility and maintain relevance, only the first 100 results per language (sorted by relevance) were reviewed. Titles and short descriptions were read to assess alignment with the PEO framework. Documents considered potentially eligible were saved using Zotero and imported into Rayyan for further screening.

Due to technical limitations during the Zotero capture process—such as broken links or incomplete metadata—only a portion of the top 100 results could be successfully saved in each language: 87 documents in English, 54 in Portuguese, and 63 in Spanish, totalling 204 records. These were subsequently integrated into the systematic screening process.

Although preliminary relevance screening was performed based on titles and descriptions at the point of collection, a complete eligibility assessment was reserved for the central review phase. The decision to limit inclusion to the first 100 results per language is consistent with established practices in systematic reviews that use Google Scholar, where search precision is inherently limited, and document types cannot be filtered directly. This approach aimed to capture high-ranking and thematically pertinent documents that may not be indexed in traditional bibliographic databases, thus enhancing the inclusivity and scope of the review.

Search query entered before applying interface filters:

English:

Field: "with all of the words"

primary care OR general practice OR family medicine OR primary healthcare OR general practitioner OR primary health care OR health professionals OR community health workers OR healthcare providers AND patient OR healthcare service users OR immigrant OR migrant OR ethnic minorities OR diverse populations OR minority groups

Field: "with at least one of the words":

climate change OR global warming OR extreme weather OR heat stress OR air pollution OR vector-borne diseases OR environmental risk OR climate-sensitive health OR climate-related health risks OR environmental change OR climate-sensitive health outcomes OR temperature-related diseases AND intercultural communication OR cultural competence OR intercultural dialogue OR cross-cultural communication OR cross-cultural dialogue OR cross-cultural OR intercultural health OR traditional knowledge OR indigenous knowledge OR cultural diversity OR community resilience

Portuguese:

Field: "with all of the words":

saúde primária OR medicina geral OR medicina familiar OR cuidados de saúde primários OR médico de medicina geral e familiar OR profissionais de saúde OR agentes comunitários de saúde OR prestadores de cuidados de saúde AND paciente OR utentes OR imigrante OR migrante OR minorias étnicas OR populações diversas OR grupos minoritários

Field: "with at least one of the words":

alterações climáticas OR mudanças climáticas OR aquecimento global OR fenómenos meteorológicos extremos OR stresse térmico OR poluição atmosférica OR doenças transmitidas por vetores OR risco ambiental OR saúde sensível ao clima OR riscos para a saúde relacionados com o clima OR alterações ambientais OR resultados de saúde sensíveis ao clima OR doenças relacionadas com a temperatura AND comunicação intercultural OR competência cultural OR diálogo intercultural OR comunicação transcultural OR diálogo transcultural OR transcultural OR saúde intercultural OR conhecimento tradicional OR saberes indígenas OR diversidade cultural OR resiliência comunitária

Spanish:

Field: "with all of the words":

primaria de salud OR medicina general OR medicina familiar OR atención primaria de salud OR médico general OR profesionales de la salud OR trabajadores comunitarios de salud OR proveedores de servicios de salud AND paciente OR usuarios de los servicios de salud OR inmigrante OR migrante OR minorías étnicas OR poblaciones diversas OR grupos minoritarios

Field: "with at least one of the words":

cambio climático OR calentamiento global OR fenómenos meteorológicos extremos OR estrés térmico OR contaminación del aire OR enfermedades transmitidas por vectores OR riesgo ambiental OR salud sensible al clima OR riesgos para la salud relacionados con el clima OR cambios ambientales OR desenlaces de salud sensibles al clima OR enfermedades relacionadas con la temperatura AND comunicación intercultural OR competencia cultural OR diálogo intercultural OR comunicación transcultural OR diálogo transcultural OR transcultural OR salud intercultural OR conocimiento tradicional OR saberes indígenas OR diversidad cultural OR resiliencia comunitaria

Search Combinations and Results

Blocks	Google Scholar		
	#Doc	Time	Records retrieved
A + B + C ENG	17100	0,06 sec.	87
A + B + C PT	136	0,12 sec.	54
A + B + C ES	277	0,13 sec.	63
**Total**			**204**

Search Combinations and Results for all Database 1st search
June 2025

Language	Search blocks	Scopus	PubMed	Web of Science	Google Scholar	RCAAP	SciELO	Other Sources
English	A + B + C	9	3	6	80			
Portuguese	A+ B+ C				55			
Spanish	A+ B+ C				17			
English	A						1610	
Portuguese	A						273	
English	A + B	702	31	315				
Portuguese	A + B	0	0	0		11		
English	A + C	2330	98	1595		0		
Portuguese	A + C	0	0	0		87		
English	B + C	4619	104	4353		0		
Portuguese	B + C					21		
English	B						143	
Portuguese	B						0	
English	C						2	
Portuguese	C						118	
Via other methods								298
**Totals**	**16 880**	**7 660**	**236**	**6 269**	**152**	**119**	**2 146**	**298**

The total number of records identified in the search, conducted in June 2025, and imported into Rayyan for screening was **16,880**.

Search Combinations and Results for all Database second search
1 April 2026

Language	Search blocks	Scopus	PubMed	Web of Science	Google Scholar	RCAAP	SciELO
English	A + B + C	6	0	1	87		
Portuguese	A+ B+ C				54		
Spanish	A+ B+ C				63		
English	A						82
Portuguese	A						19
English	A + B	161	11	57			
Portuguese	A + B	0	0	0		3	
English	A + C	321	28	134		0	
Portuguese	A + C	0	0	0		5	
English	B + C	284	28	939		0	
Portuguese	B + C					3	
English	B						25
Portuguese	B						0
English	C						2
Portuguese	C						18
**Totals**	**2,331**	**772**	**67**	**1,131**	**204**	**11**	**146**

The total number of records identified in the search, conducted in April 2026, and imported into Rayyan for screening was **2,331**.

Total of the Search Combinations and Results for all Database

Language	Search blocks	Scopus	PubMed	Web of Science	Google Scholar	RCAAP	SciELO	Other Sources
English	A + B + C	15	3	7	167		0	
Portuguese	A+ B+ C				109		0	
Spanish	A+ B+ C				80		0	
English	A						1692	
Portuguese	A						292	
English	A + B	863	42	372				
Portuguese	A + B	0	0	0		14		
English	A + C	2651	126	1729		0		
Portuguese	A + C	0	0	0		92		
English	B + C	4903	132	5292		0		
Portuguese	B + C					24		
English	B						168	
Portuguese	B						0	
English	C						4	
Portuguese	C						136	
Via other methods								298
**Totals**	**19,211**	**8,432**	**303**	**7,400**	**356**	**130**	**2,292**	**298**

The total number of records identified and imported into Rayyan for screening was **19,211.**
