# Supplementary material for: Primary healthcare, climate-related health impacts and intercultural dialogue: bibliometric evidence of a fragmented research field
Source: Front Health Serv. 2026 Sep 30;6:1909093. doi: 10.3389/frhs.2026.1909093 (PMC13662001; doi:10.3389/frhs.2026.1909093)
Supplement: Supplementary file 3 [file Datasheet3.pdf]

Supplementary File S3: Screening Label Book

Primary healthcare, climate-related health impacts and intercultural dialogue: bibliometric evidence of a fragmented research field

Authors: Nidia Ponte, Fátima Alves, Diogo Guedes Vidal

Journal: Frontiers in Health Services

Article type: Systematic Review

PROSPERO registration: CRD420251038912

OSF registration: 10.17605/OSF.IO/AXYDW

This screening label book was used to support screening and eligibility decisions according to the PEO framework defined in the review protocol.

Population (P) refers to primary healthcare physicians, particularly general and family medicine physicians/general practitioners, and adult patients or service users from diverse sociocultural backgrounds.

Exposure (E) refers to the health impacts of climate change, addressed directly or indirectly, including phenomena such as heat stress, extreme weather events, food insecurity, forced displacement, or other forms of ecological vulnerability. This also includes how these exposures are recognised and responded to through face-to-face communication between healthcare professionals and patients.

Outcome (O) refers to intercultural dialogue strategies developed and/or implemented, including cultural competence education and equity-oriented approaches, that contribute to climate adaptation and community resilience and are relevant to the doctor–patient relationship in primary healthcare, or demonstrably transferable to it.

Although the protocol allowed the inclusion of records addressing at least two of the three PEO dimensions, the E + O combination was not used as an independent inclusion label. This is because the Outcome was operationally defined as intercultural dialogue strategies relevant to the doctor–patient relationship in primary healthcare, or demonstrably transferable to it. Therefore, when O was present according to the review definition, a Population/context component was necessarily explicit, implicit, or transferable. Records addressing climate change and interculturality without a clear link to healthcare professionals, patients/service users, or clinical communication were not coded as E + O, but were excluded as E only, O only, or PEO-mismatch, depending on their content.

Records were included when they addressed P + E + O, P + E, or P + O according to the operational definitions below. Records addressing only one dimension, or none of the eligible PEO combinations, were excluded.

Label	Decision	Description in English
P + E + O	Include	Studies that:<br>**P** = involve primary healthcare physicians, particularly general and family medicine physicians/general practitioners, and adult patients or service users from diverse sociocultural backgrounds.<br>**E** = address the health impacts of climate change, directly or indirectly, including phenomena such as heat stress, extreme weather events, food insecurity, forced displacement, or other forms of ecological vulnerability.<br>**O** = analyse intercultural dialogue strategies developed and/or implemented, including cultural competence education and equity-oriented approaches, that contribute to climate adaptation and community resilience and are relevant to the doctor–patient relationship in primary healthcare, or demonstrably transferable to it.
P + E	Include	Studies that:<br>**P** = involve primary healthcare physicians, particularly general and family medicine physicians/general practitioners, and adult patients or service users from diverse sociocultural backgrounds.<br>**E** = address the health impacts of climate change, directly or indirectly, including phenomena such as heat stress, extreme weather events, food insecurity, forced displacement, or other forms of ecological vulnerability.
P + O	Include	Studies that:<br>**P** = involve primary healthcare physicians, particularly general and family medicine physicians/general practitioners, and adult patients or service users from diverse sociocultural backgrounds.<br>**O** = analyse intercultural dialogue strategies developed and/or implemented, including cultural competence education and equity-oriented approaches, that are relevant to the doctor–patient relationship in primary healthcare, or demonstrably transferable to it.
P	Exclude	Studies that involve only primary healthcare professionals, particularly general and family medicine physicians/general practitioners, and/or adult patients or service users from diverse sociocultural backgrounds.<br>Excluded because they address only the population or healthcare-provider dimension, without climate-related exposure or intercultural dialogue outcome.

E	Exclude	Studies focused only on the health impacts of climate change, addressed directly or indirectly, including issues such as heat stress, extreme weather events, food insecurity, forced displacement, or other forms of ecological vulnerability, but without involving healthcare professionals, patients/service users, or communication between them.<br>Excluded because they address only the climate-related health impact dimension, without the Population or Outcome dimensions required by the review.
O	Exclude	Studies that deal only with interculturality, intercultural dialogue, cultural competence, or related concepts in a broad sense and without a clear connection to clinical practice, the doctor–patient/professional–service user relationship, or transferability to primary healthcare.<br>Excluded because they address only the interculturality/intercultural dialogue dimension without meeting the review's operational definition of Outcome.
PEO-mismatch	Exclude	Studies that do not adequately address any eligible combination of the three central dimensions of the review: health professionals/patients or service users (P), climate change and related health impacts (E), and intercultural dialogue strategies relevant to clinical communication (O).<br>Excluded because they do not fit within the conceptual scope of the review.
Duplicate	Exclude	Duplicate record.
Outside the eligible period	Exclude	Publications whose date falls outside the time interval defined for the review.
Full text not available	Exclude	Restricted-access text: after searches in B-On (UAb), Scopus (UAb), ResearchGate and Google, the full text of the document could not be located. In the absence of full-text access, eligibility could not be assessed according to the review criteria.
Wrong publication type	Exclude	Eligible records include articles in scientific journals, including original research and review articles, systematic reviews, scoping reviews, book chapters, books, conference papers, theses and dissertations.<br>Excluded publication types include editorials, letters, comments, notes, short surveys, abstracts and errata, since these rarely present peer-reviewed content, in-depth analysis, or original data.

Wrong language	Exclude	Eligible languages are Portuguese, English and Spanish. All other languages are not eligible.
Missing abstract and title	Exclude	After several searches, it was not possible to find the title and abstract of the record indicated by the databases.
Missing abstract	Exclude	After several searches, it was not possible to find the abstract of the record indicated by the databases.
