# Supplementary material for: Primary healthcare, climate-related health impacts and intercultural dialogue: bibliometric evidence of a fragmented research field
Source: Front Health Serv. 2026 Sep 30;6:1909093. doi: 10.3389/frhs.2026.1909093 (PMC13662001; doi:10.3389/frhs.2026.1909093)
Supplement: Supplementary file 4 [file Datasheet4.pdf]

Supplementary File S4: Supplementary Tables

Primary healthcare, climate-related health impacts and intercultural dialogue: bibliometric evidence of a fragmented research field

Authors: Nidia Ponte, Fátima Alves, Diogo Guedes Vidal

Journal: Frontiers in Health Services

Article type: Systematic Review

PROSPERO registration: CRD420251038912

OSF registration: 10.17605/OSF.IO/AXYDW

Supplementary Table S1. Illustrative examples of duplicate pairs comparing Scopus and Web of Science metadata

Example	Internal record ID	Short title	Matching criterion	Field compared	Scopus record	Web of Science record	Record retained
1	rayyan-272062940	*Cultural competence in health visiting practice*	Normalised title + author + journal + volume/issue/pages	URL/access link	Present	Missing	Scopus
2	rayyan-272070322	*Nesor Annim, Niteikapar…*	Normalised title + author + journal + volume/issue/pages	URL/access link	Present	Missing	Scopus
3	rayyan-272046594	*Assessing the ripple effects of natural disasters on healthcare systems*	DOI + title + author + journal + volume/issue/pages	URL/access link	Present	Missing	Scopus
4	rayyan-272068944	*Bodies of Knowledge…*	Normalised title + author + journal + volume/pages	URL/access link	Present	Missing	Scopus

Note. Examples were selected to illustrate the duplicate-management rule applied when the same publication was retrieved from both Scopus and Web of Science. Duplicate matching was based on DOI where available or, when DOI was absent, on normalised title, first author, journal/source and volume, issue or page information. In these examples, the Scopus record was retained because it provided a direct operational URL/access link, which facilitated retrieval and subsequent screening. These examples are illustrative and do not represent all duplicate pairs.

Supplementary Table S2. Outcome of the approximately 10% title-and-abstract screening verification by source

Reassessment outcome	RCAAP	Google Scholar	PubMed	Other sources	SciELO	Web of Science	Scopus	Total
Records provisionally excluded at first title-and-abstract screening	117	322	216	211	1,666	3,185	7,308	13,025
Records reassessed, n (% of provisionally excluded records)	12 (10.3%)	32 (9.9%)	22 (10.2%)	22 (10.4%)	171 (10.3%)	334 (10.5%)	764 (10.5%)	1,357 (10.4%)
Confirmed as excluded after reassessment	12	32	13	22	171	334	763	1,347
Reclassified as potentially eligible after reassessment	0	0	9	0	0	0	1	10
Final title-and-abstract exclusions after verification	117	322	207	211	1,666	3,185	7,307	13,015

Supplementary Table S3. Parameters used for the keyword co-occurrence analysis

Parameter	Decision
Analytical corpus	Full-text assessed corpus
Number of documents	745
Software	VOSviewer
Type of analysis	Co-occurrence analysis
Unit of analysis	Keywords
Keyword fields	Database keywords
Counting method	Full counting
Minimum occurrence threshold	3
Number of keywords included in the map	344
Number of clusters	11
Visualisation type	Network visualisation
Clustering	VOSviewer default clustering algorithm
Data cleaning	A VOSviewer thesaurus was applied to remove administrative tags and source-related labels, and to normalise selected lexical, spelling and formatting variants
Harmonisation criteria	Selected variants were standardised only when they referred to the same analytical term; no extensive conceptual recoding of keywords was performed
Terms removed	Administrative screening tags, database/source labels, access-status labels, author-contact notes and selected low-interpretability , and geographical names used only as location descriptors were removed.
Robustness check	A fractional-counting map was inspected as a sensitivity check. The full-counting map was retained for the main figure because it provided a broader and more interpretable representation of the thematic structure.

Note. The thesaurus was used primarily to reduce technical noise introduced during record management and database export. It was not intended to impose a new thematic taxonomy on the corpus. The keyword map file exported from VOSviewer included 344 keywords and 11 clusters. Geographical names were removed when they functioned only as location descriptors, because geographical distribution was analysed separately; population, cultural, ethnic or identity-related terms were retained when analytically relevant to sociocultural diversity, interculturality and health equity.

Supplementary Table S4. Leading country-level study contexts in the curated bibliometric corpus

Rank	Region	Study-context category	n
-	Not specified	Not specified	109
-	Multiple countries	Multiple countries	77
1	North America	United States of America	98
2	South America	Brazil	93
2	Oceania	Australia	59
3	North America	Canada	40
4	Europe	Portugal	30
5	Sub-Saharan Africa	South Africa	21
6	Europe	United Kingdom	21
7	Europe	Spain	18
8	Europe	Netherlands	12
9	South America	Colombia	11
10	Central America	Mexico	11
11	Europe	Italy	10
12	South America	Chile	9
13	Europe	Belgium	8
14	South America	Peru	8
15	Europe	Sweden	7
16	Europe	Germany	6
17	Caribbean	Cuba	5
18	Europe	Denmark	5
19	East and Southeast Asia	Indonesia	5
20	Europe	Norway	5
